# SARS-CoV-2 and other human coronavirus show genome patterns previously associated to reduced viral recognition and altered immune response

**DOI:** 10.1038/s41598-021-90278-4

**Published:** 2021-05-21

**Authors:** Giovanni Franzo

**Affiliations:** Department of Animal Medicine, Production and Health (MAPS), University of Padua, Viale dell’Università 16, 35020 Legnaro, Padua Italy

**Keywords:** Microbial genetics, Virology, Immune evasion

## Abstract

A new pandemic caused by the betacoronavirus SARS-CoV-2 originated in China in late 2019. Although often asymptomatic, a relevant percentage of affected people can develop severe pneumonia. Initial evidence suggests that dysregulation of the immune response could contribute to the pathogenesis, as previously demonstrated for SARS-CoV. The presence of genome composition features involved in delaying viral recognition is herein investigated for human coronaviruses (HCoVs), with a special emphasis on SARS-CoV-2. A broad collection of HCoVs polyprotein, envelope, matrix, nucleocapsid and spike coding sequences was downloaded and several statistics representative of genome composition and codon bias were investigated. A model able to evaluate and test the presence of a significant under- or over-representation of dinucleotide pairs while accounting for the underlying codon bias and protein sequence was also implemented. The study revealed the significant under-representation of CpG dinucleotide pair in all HcoV, but especially in SARS-CoV and even more in SARS-CoV-2. The presence of forces acting to minimize CpG content was confirmed by relative synonymous codon usage pattern. Codons containing the CpG pair were severely under-represented, primarily in the polyprotein and spike coding sequences of SARS-CoV-2. Additionally, a significant under-representation of the TpA pair was observed in the N and S region of SARS-CoV and SARS-CoV-2. Increasing experimental evidence has proven that CpG and TpA are targeted by innate antiviral host defences, contributing both to RNA degradation and RIG-1 mediated interferon production. The low content of these dinucleotides could contribute to a delayed interferon production, dysregulated immune response, higher viral replication and poor outcome. Significantly, the RIG-1 signalling pathway was proven to be defective in elderlies, suggesting a likely interaction between limited viral recognition and lower responsiveness in interferon production that could justify the higher disease severity and mortality in older patients.

## Introduction

The family *Coronaviridae* includes a large group of enveloped, positive-stranded RNA viruses further classified in 4 genera *Alpha-*, *Beta-*, *Gamma-*, *and Deltacoronavirus*.

The coronavirus genome is of extraordinarily large size compared to other RNA viruses, ranging between about 26–32 kilobases^1^. The 5′ two-thirds of the genome code for two polyproteins, pp1a and pp1ab, which are then proteolytically cleaved in several nonstructural proteins. Production of the pp1ab requires the translating ribosome to change the reading frame at the frameshift signal that bridges ORF1a and ORF1ab^2^. The final one-third of the genome encodes a set of four structural protein genes, in the order of spike (S), envelope (E), membrane (M) and nucleocapsid (N). Besides, several accessory ORFs are also interspersed along the structural protein genes and the number and location varies among CoV species^3^.

Until 2019, six human coronaviruses (HCoVs) have been identified, including the two alpha-CoVs HCoV-NL63 and HCoV-229E and the beta-CoVs HCoV-OC43, HCoV-HKU1, severe acute respiratory syndrome-CoV (SARS-CoV) and Middle East respiratory syndrome-CoV (MERS-CoV)^4^. All these viruses cause mainly respiratory signs and HCoV-NL63, HCoV-229E, HCoV-OC43 and HCoV-HKU1 have been associated to the common cold and mild, upper respiratory tract infections, although lower respiratory tract involvement and more serious respiratory disease can occur in children, elderly and persons with underlying illness^5,6^.

Even if HCoVs have been identified for decades, their clinical importance was limited until the emergence of the two epidemic SARS-CoV and MERS-CoV viruses. SARS-CoV originated from bats species through a likely passage in carnivores in 2003 and affected more than 8000 people, with 916 deaths (case fatality approximatively 10%) in 29 countries before being eradicated by global control efforts^7^. MERS-CoV, incidentally identified in 2012, was then proven to regularly infect humans who acquire the infection from dromedary camels, although bats were again identified as the original reservoir. Since the first reports, more than 2000 cases have been described, with a case fatality higher than 30%^4^.

In late 2019, a new pandemic HCoV (SARS-CoV-2) emerged in the city of Wuhan in Hubei province, and despite unprecedented control measures implemented in China and other countries, SARS-CoV-2 has reached a worldwide distribution^8^. At the moment of writing, 212 countries have reported SARS-CoV-2 detection, for a total of 1,353,361 cases and 79,235 deaths (https://www.who.int/emergencies/diseases/novel-coronavirus-2019). However, the lack of diagnostic assays, especially in the initial epidemic phase, and of properly performed epidemiological studies hampers the comprehension of the actual infection prevalence. Mathematical simulation based on the data available from 11 European countries estimates that between 7 and 43 million individuals have been infected with SARS-CoV-2 across all considered countries by the end of March, representing between 1.88% and 11.43% of the population, with peaks of 15% in some countries^9^.

While most of the infected people had an asymptomatic infection or mild disease, 20% of ill people develop severe disease, with an overall case fatality rate of more than 3% among confirmed cases. Several factors seem to affect the risk of severe disease and mortality, including gender, co-morbidities and age^10^.

Although distinct, MERS-CoV, SARS-CoV and SARS-CoV-2 share some common clinical and pathogenic features^11^. SARS-CoV and MERS-CoV severe to fatal cases were predominantly characterized by interstitial pneumonia, edematous lungs with acute diffuse bronchial and alveolar damage, coupled with increased monocyte, macrophage, and neutrophil infiltration in the lungs^11^. Elevated levels of proinflammatory cytokines and chemokines were also identified, indicative that both virus‐induced cytopathic effects and immunopathology due to “cytokine‐storm” are likely contributing to disease severity and poor prognosis^12^. High levels of proinflammatory cytokines were measured in SARS patients with severe disease compared to uncomplicated cases.

Initials studies on SARS-CoV-2 evidenced overlapping signs and lesions and patients requiring intensive care (IC) showed higher plasma levels of IL‐2, IL‐7, IL‐10, GSCF, IP10, MCP1, MIP1A, and TNF‐α than non‐IC patients^13^.

Coronaviruses have evolved different mechanisms to alter cell signalling, delay innate immunity and deregulate immune effectors. Several coronavirus proteins have been demonstrated to interact at different levels of, but not limited to, interferon I (INF-I) pathways^14–16^. Accordingly, delayed interferon production and consequent dysregulated innate immune response have been linked to lethal infection in experimental mice model^17^.

A rising amount of evidence suggests that besides proteins, also viral genome composition could be involved in minimizing viral recognition and therefore delay the activation of an effective immune response. Several studies have demonstrated the presence of a relevant genomic signature in dinucleotide frequencies of different organisms^18^. This evidence has been reported also for viral genomes and could be the result of pressures induced by the “host environment” and its immune defences^19^. Interestingly, even the virome of different environments is featured by a distinct pattern, which confirms the direct or indirect effect of environmental conditions on organism genome composition^20^.

Codon bias is another phenomenon affecting the fitness of the genome without a direct impact on proteins. Because of the degeneracy of the genetic code, most amino acids are encoded by two to six different codons^21,22^. However, different synonymous codons are used with different frequencies among organisms or even among tissues of the same organism^23^. Being intracellular obligate parasites, viruses must be able to both efficiently exploit the cell synthetic machinery and avoid recognition mechanisms. Accordingly, the virus-host mimicry in terms of codon bias and genome composition as well as the progressive viral adaptation after a host jump has been occasionally reported^24–27^.

The present study investigates the genome composition of HCoVs, with a special emphasis on SARS-CoV-2 to evaluate the presence of patterns that could contribute in innate immunity deregulation, favouring viral replication and therefore infection spreading and disease severity.

## Results

### Genome composition

Although a different nucleotide percentage was identified among genes and species, some common features were present. Particularly, G and C nucleotide were significantly less present than A and T, independently from the viral species and genes (Supplementary figure 1 and table 1). Overall, the G + C content decreased from the first to third codon position. The envelope protein was a significant exception, being featured by a higher GC3 content than CG2. The same could be observed in the matrix protein of HCoV-229E, MERS-CoV and SARS-CoV.

**Figure 1:**
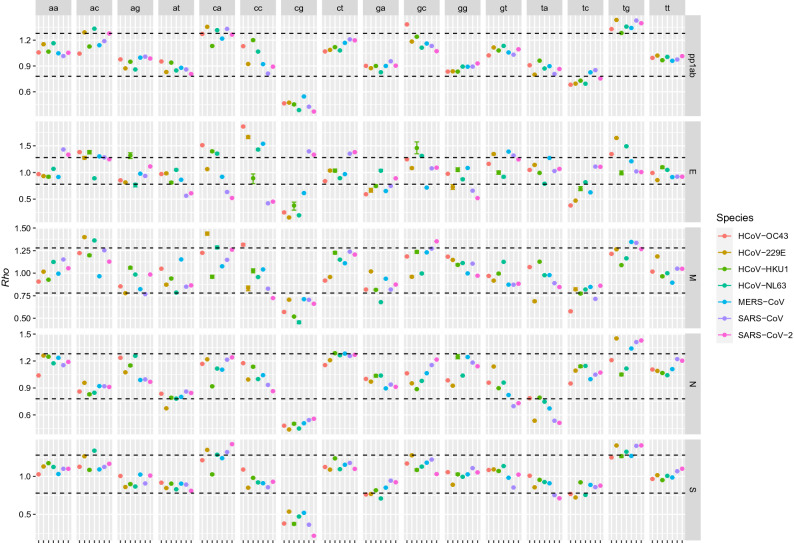
Mean (point) and 95% confidence intervals (errorbar) of *Rho* statistic calculated for each dinucleotide pair. Different gene-species pair are reported in different cells. Dashed lines represent the cut-offs defined by Karling et al. (1998).

The analysis of dinucleotide odds ratio revealed a remarkable heterogeneity. However, all HCoVs demonstrated a relevant under-representation of the CpG pair. SARS-CoV-2 was the species with the lowest CpG *Rho* in the pp1ab and especially in S coding sequence (CDS). On the other hand, SARS-CoV-2 and SARS-CoV were the only species where this dinucleotide was over-represented in the E coding gene (Fig. 1). Among the HCoVs inducing severe disease, MERS-CoV demonstrated the overall less biased usage of this dinucleotide. The TpA pair was also significantly under-represented in HCoV-229E, SARS-CoV and SARS-CoV-2 (and to a lesser extent in MERS-CoV) in the nucleocapsid, and slightly under-represented in the S gene of SARS-CoV and SARS-CoV-2.

Most of the other dinucleotide pairs were within the expected ranges, although TpG was over-represented in pp1ab of all viruses and in species-specific fashion for other genes.

**Figure 2:**
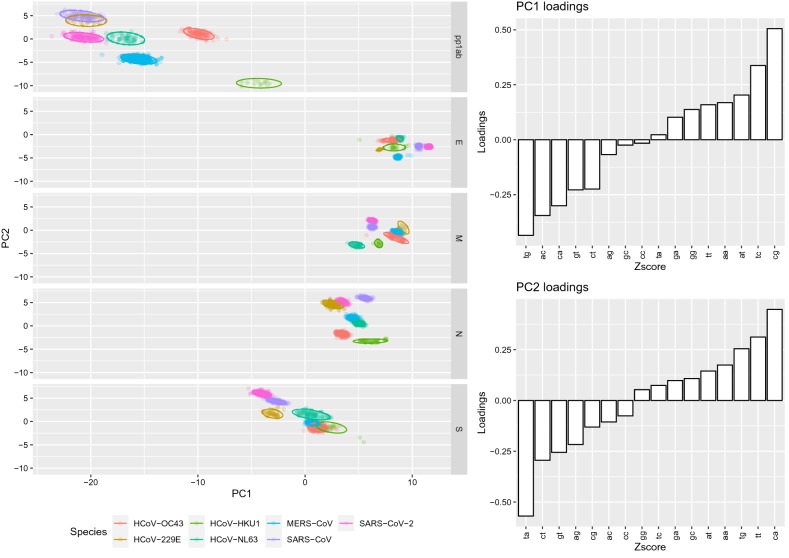
Scatter plot based on the first two components of the PCA performed on Zscore (**a**). The different proteins are reported in separate rows while viral species are color-coded. The PC1 and PC2 loadings are reported in the right insert.

The Zscore confirmed the observed scenario, reinforcing that the CpG was significantly under-represented even accounting for amino acid composition and codon bias (Supplementary figure 2). However, the CpG odds ratio of SARS-CoV and SARS-CoV-2 fall within the expected range in the E genes, differently from what observed by crude *Rho* estimation. On the other hand, an over-representation of ApC, CpA and TpG pair was observed in the pp1ab and S of essentially all viruses, being the S of HCoV-NL63 the main exception. The first two principal components (PC) of PCA based on Zscores explained 78% and 8.9% of the overall variance and were therefore maintained to explore the data. A clear separation could be observed among proteins while viral species demonstrated a largely overlapping distribution (Supplementary figure 3). However, within each gene, viral HCoVs could be differentiated (Fig. 2). PC1 loadings inspection shoved that CpG had the highest positive correlation with this principal component. The HCoVs proteins distribute along PC1 axis following a length-dependent pattern. pp1ab located at the most negative extreme, followed by S, N, M and E. Therefore, a negative correlation could be observed between coding sequence length and CpG content.

Within each protein, pp1ab and S coding gene of SARS-CoV and SARS-CoV-2 locate at the most negative extreme of PC1. SARS-CoV and SARS-CoV-2, together with HcoV-229E, had lower PC1 values compared to other species in the M also, while only SARS-CoV-2 in the N gene. On the contrary, they showed the highest values in the E coding sequence.

In the second PC (PC2), CpA and TpA showed the highest positive and negative correlation, respectively (Fig. 2 and Supplementary figure 3), while CpG showed a certain negative correlation. SARS-CoV-2 was associated to higher PC2 values in S (together with SARS-CoV) and N (together with HCoV-229E and SARS-CoV). Slightly positive to neutral values featured SARS-CoV-2 in pp1ab, M and E gene, respectively.

### Codon bias

RSCU analysis highlighted a similarly heterogeneous pattern. However, the under-representation of codon containing the CpG dinucleotides was a common feature, affecting all viral proteins and viral species (Supplementary figure 4). However, SARS-CoV-2 only had all these codon under-represented (or, to a lesser extent, normally represented) in pp1ab gene, while in the S gene also SARS-CoV and HCoV-OC43 shared this feature. On the contrary, a higher number of CpG rich codons was observed in the E coding gene.

**Figure 3:**
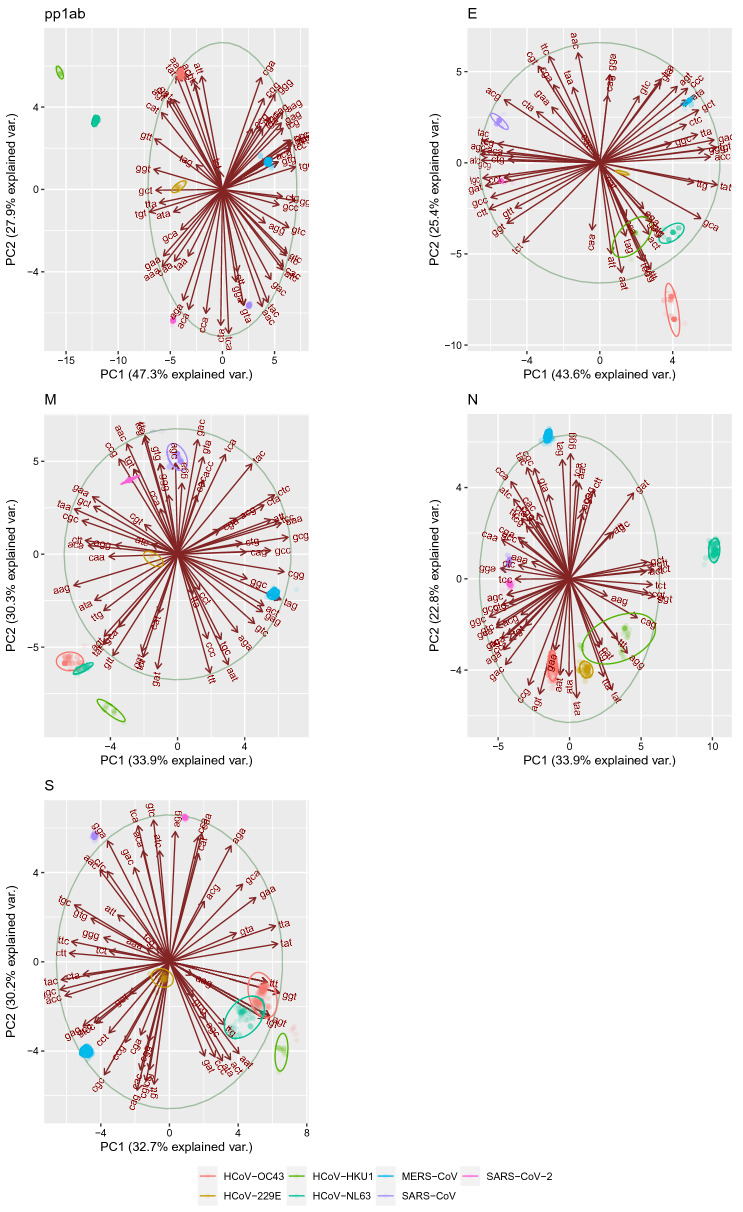
Scatter plot based on the first two components of the PCA performed on RSCU. The different proteins are reported in separate cells while viral species are color-coded. The loadings are represented as arrows and the corresponding correlation circle has been reported. The 95% confidence ellipses around clusters are also reported. A more detailed representation is provided in Supplementary figures 5–9.

PCA performed on RSCU highlighted a less clear overall pattern, featured by a lower variance explained by each PC. However, when performed at the individual gene level, the differentiation among species could still be achieved. Independently on the gene, most of the codons with CpG were typically highly correlated with at least one of the PC (Fig. 3 and Supplementary figures 5–9). In pp1ab, with only one exception, all CpG rich codons were positively correlated with PC1 and PC2 (Supplementary figure 5). SARS-CoV-2 was the only viral species with highly negative values in both PCs. On the contrary, MERS-CoV located in the quadrant featured by higher positive values. Similarly, SARS-CoV and SARS-CO-2 had negative PC2 values, while most CpG codons were positively correlated with this component (Fig. 3 and Supplementary figure 5). In the S region, most of CpG rich codons were negatively correlated to PC1 and PC2. SARS-CoV-2 located in the positive quadrant of both PC, while SARS-CoV had positive values in the PC2 and negative ones in PC1 (Supplementary figure 6). In the N, M and E genes, SARS-CoV-2 located in regions defined by a neutral to positive association with CpG content, although in M and N genes, CpG containing codons correlation with PC was less polarized (Supplementary figures 7–9).

**Figure 4:**
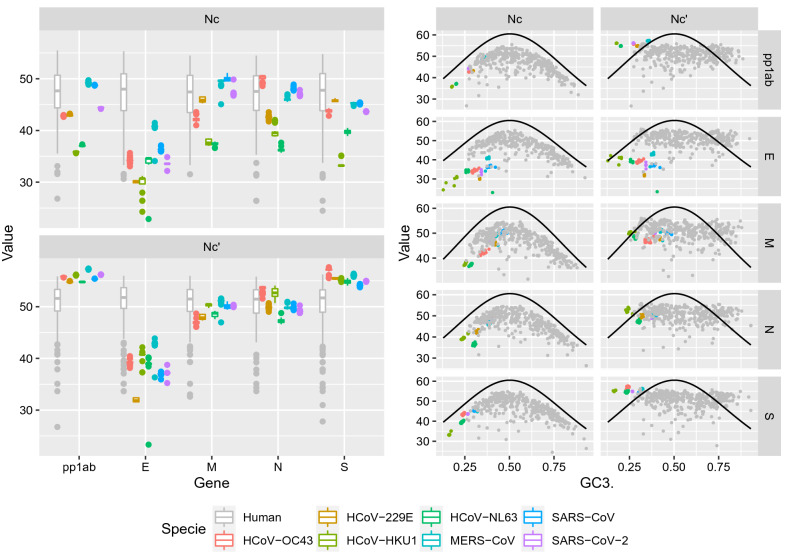
Left figure: Boxplot reporting the Nc and Nc′ values of HCoV species (color-coded). For comparison, the host value distribution (in grey) is reported nearby each gene. Right figure: Scatterplot reporting the relationship between Nc and Nc′ and GC3 content of HCoVs coding sequences. HCoVs proteins have been color-coded while the host lung genes have been reported in grey. The line representing the expected Nc values, which would result from GC3 composition being the only factor influencing the codon usage bias, has been superimposed.

A differential codon usage among proteins could be confirmed by effective codon usage analysis. E gene showed an overall more biased codon usage (lower Nc) compared to other viral proteins and the host coding sequences. Among HCoVs, MERS-CoV, SARS-CoV and SARS-CoV-2 showed a higher Nc, fully overlapping with the host one in all but E coding regions. HCoV-HKU1, HCoV-NL63 and to a lesser extent HCoV-OC43 and HCoV-229E had a lower Nc, distinct from the host, although exceptions were present for specific species-gene pairs (Fig. 4).

Nc′ was significantly higher compared to Nc, leading to values even greater than the human genes ones in the pp1ab and S coding region, testifying that viral genome nucleotide composition had a relevant effect on codon bias. However, even after accounting for this component, effective codon number deviated from what expected based on GC3 composition only (Fig. 4).

## Discussion

Epidemic HCoVs, including SARS-CoV, MERS-CoV and SARS-CoV-2, although targeting several cell lines, are featured by a higher involvement of the lower respiratory tract compared to other HCoVs and can, therefore, be responsible for severe pneumonia^11,28^. Besides direct damages due to viral replication, the dysregulation of the host immune response can induce immune cell infiltration and cytokine storm, leading to severe disease occurrence and fatalities. In mouse experimental models, delayed IFN-I signalling was associated with accumulation of pathogenic inflammatory monocyte-macrophages in the lung and elevated expression of several pro-inflammatory cytokines and chemokines and consequent lung immunopathology^17^. However, IFN-I administration before the viral replication peak protected mice from clinical disease^17^. Therefore, the prompt innate response can limit viral replication and control the downstream activation of immune-mediated damages.

The analysis of genome composition of HCoVs revealed a remarkable bias in several dinucleotide pair usage, as testified by several *Rho* lower than the 0.78 and 1.23 cut-offs proposed by Karlin et al. (1998) (Fig. 1). However, these thresholds can be considered accurate for long sequences only^18^. Additionally, dinucleotide frequency could be affected by codon bias and by amino acid composition, imposed by protein functional constraints. To deal with this issue, a permutation approach, reshuffling the synonymous codons along the protein (i.e. without affecting the overall codon usage bias and protein structure), was implemented to normalize the *Rho* value through random sequences generation, allowing statistical testing.

Both *Rho* and Zscore highlighted a significant CpG under-representation compared to what expected by chance and nucleotide frequency alone, similarly to what reported by other authors for other RNA viruses^19,29^ and HCoVs^30,31^. This pair is well-known to be underrepresented in eukaryotic genomes since cytosine in CpG dinucleotides is easily methylated and tend to spontaneously deaminate into thymine. However, methylation does not seem to occur in RNA viruses, which use their synthetic apparatus for genome replication and transcription^32^. Higher stacking energy associated to CpG could lead to stronger secondary structures in ssRNA viruses and affect transcription and translation efficiency, as proposed for ssDNA viruses^33^ and recently for SARS-CoV-2 also^34^. However, the corresponding GpC and other pairs featured by high thermal energy were normally represented in the same genes (Fig. 1), contradicting this hypothesis. Unmethylated CpG DNA is a well-known target of the pattern recognition receptor (PRR) Toll-like receptor 9 (TLR-9) in mammals and is thus involved in innate immune response activation, thus explaining the tendency of DNA viruses to reduce their CpG content. Although different pattern recognition receptors like TLR-3, TLR-7, TLR-8, RIG-I and MDA5 were recognized to target viral RNA, none of those specifically recognizes CpG motifs^14^. However, Atkinson et al. demonstrated that experimentally increasing the CpG content in some RNA viruses led to attenuation, lower replication rate and low competitive fitness relative to wild-type^35^. Takata et al. proved that the zinc-finger antiviral protein (ZAP) selectively binds to sequences containing CpG dinucleotide and HIV strains whose CpG content has been modified are defective in the normal cells but able to replicate in ZAP defective ones^36^. Particularly, ZAP was reported to interact with viral RNA and lead to its degradation^37,38^. Additionally, a shorter ZAP isoform (ZAPS) has a regulatory activity on RIG-1 signaling, strengthening the RIG-I-mediated induction of type I interferons and other inflammatory cytokines^39^. In fact, its actual role in antiviral innate immune responses against influenza virus and Newcastle disease virus was experimentally proven^39^.

Therefore, HCoVs CpG content is likely under strong selective constraints to minimize viral recognition, degradation and/or activation of host innate immunity^30^. The observed CpG ratio would thus be part of a broader HCoVs escape mechanism, likely in concert with viral proteins^14^. The ZAP induced selective pressure has actually been proposed as a SARS-CoV-2 evolution driving force, suggesting that SARS-CoV-2 may have evolved in a host with high ZAP expression^30^. The depletion in CpG content was negatively correlated to coding sequence length (Fig. 2) and a significant (*p* < 0·001) negative relationship was identified between CpG ratio (i.e. CpG count ÷ CDS length) and gene length (Supplementary figure 10 and Supplementary table 2), particularly for SARS-CoV and SARS-CoV-2. A stronger selective pressure acting on mRNAs containing the higher absolute amount of CpG can be hypothesized. Significantly, SARS-CoV and SARS-CoV-2 are the HCoVs featured by the more pronounced bias^30^, particularly in the longest CDS, coding for pp1ab and S. These genome features could severely impair viral recognition in the early infection phases when viral nucleic acids are the more abundant viral pathogen-associated molecular patterns (PAMPs), and when the inhibitory effect of viral proteins on cellular defense mechanisms is still modest. This could be associated with limited or delayed INF production, higher viral replication and immune response deregulation, leading to a poor outcome. SARS-CoV and SARS-CoV-2 also displayed a lower TpA content, which is frequently reported to be under-represented in eukaryotic genomes^22^. TpA recognition in viral RNA sequences is described as a vertebrate immune response mechanism and other human viruses like West Nile Virus (WNV) and Hepatitis C virus (HCV) are known to be recognized by RNase L^40,41^. Accordingly, artificial increase in TpA content resulted in viral attenuation, although less marked compared to CpG^35^. This feature could further promote immune evasion, enhancing viral replication. On the contrary, MERS-CoV had a lower degree of these dinucleotides under-representation. If this could be associated to a more intense immune response, severe disease and case fatality rate, as proposed for the original 1918 H1N1 influenza virus and the recent H5N1 avian viruses (featured by a higher CpG content)^29^, would require further investigations.

The strong constraints acting on CpG were confirmed by RSCU analysis, which appeared greatly affected by the underling dinucleotide bias. In fact, the under-representation of codons containing the CpG pair was a common feature of HCoVs. Nevertheless, it was particularly evident in the SARS-CoV-2 pp1ab and S coding regions. The pattern was progressively less marked in other genes, in a CDS length-dependent fashion. Particularity, the E gene was proven in countertrend. If other factors besides gene length are involved (e.g. mRNA transcription level and timing), remains to be established. The CpG enrichment in SARS-CoV-2 E coding gene could be involved in necessary secondary structure, stimulation of ZAP activity (similarly to other viruses) intentionally triggering NF-kB or translation regulation strategy by enhancing a higher RNA degradation compared to other viral transcript^31^. Alternatively, it has been reported that the body TRS sequence of the E gene is relatively weak in SARS-CoV-2 and SARS-CoV, suggesting that this subgenomic mRNA may be of relatively low abundance and thus, in combination with the short sequence length, the high CpG frequency might be inconsequential^31^.

In addition to comorbidities, age is one of the most relevant risk factors for severe disease occurrence and death. A decreased efficiency of several components of the immune system was proven in elderlies. Among those, deficiency in the induction of type I interferon (IFN) was described in response to Influenza A virus infection in older patients^42^. Of note, both direct and indirect defects acting on the RIG-1 pathways occurs. The direct effects are ascribable to increased basal proteasomal degradation of the adaptor protein tumor necrosis factor receptor–associated factor 3 (TRAF3), which impairs the primary induction of IFN expression downstream of RIG-I signaling. The indirect ones are due to the impaired expression of the transcription factor IRF8 in older people, which is further exaggerated by the initial defects in IFN secretion and leads to a marked decrease in positive feedback amplification of the IFN response^43^. It is therefore tempting to speculate that an interaction between a defective RIG-1 signaling pathway and low RIG-1 activation due to poor viral recognition could exacerbate the delay and effectiveness of INF production in older patients, contribution to a poor outcome. Therapeutic strategies acting on this axis could boost antiviral responses to SARS-CoV-2, and other infection as well, reducing morbidities in ageing population.

Clearly, dinucleotide composition alone cannot explain the different epidemiological and clinical features of HCoVs. Receptor and tissues tropism, as well as differential viral protein function and interaction with host ones, play a major role in the final outcome. Interestingly, HCoVs responsible for severe disease demonstrated a higher effective number of codons, overlapping the host lung one, which could suggest a higher ability to exploit the cell replicative machinery. In fact, while genome composition and dinucleotide frequency preeminently affected RSCU, a residual deviation from expectations was observed even after accounting for these factors. Thus, other forces are likely acting directly on codon bias.

The present study demonstrates a severe under-representation of some dinucleotide pairs, CpG and to a lesser extent TpA, in SARS-CoV and even more in SARS-CoV-2. Since these motifs have been proven to be the target of PRRs, the SARS-CoV-2 genome features are likely to contribute in preventing viral recognition in the early infection phases, potentially leading to poorly effective and dysregulated immune response^13^, as demonstrated for SARS-CoV. These effects could be magnified in elderly people where the components of the involved signaling pathway are already defective. The underlying biological processes could, therefore, be considered a primary therapeutic target aimed to reactivate and boost patient response to viral infection. Additionally, these pieces of evidence could contribute to the development of genetically engineered vaccines, like RNA vaccines, able to elicit a strong initial innate immunity without affecting the protein phenotype and therefore their structure and immunogenicity.

## Material and methods

### Dataset

The broadest available collection of pp1a, E, M, N and S complete coding sequences obtained from HCoVs was downloaded using the ViPR on-line tool (accessed 27/03/2020)^44^. In-house developed Python scripts were used for gene and features extraction, benefiting from the Biopython library functions^45^. Different datasets were created for each coding DNA sequence (CDS) and sequences with unknown nucleotide, frameshift mutations, premature stop codons or derived from experimental models were excluded from further analysis.

### Viral genome composition analysis

For each sequence, the following statistics were obtained: the content of each nucleotide, total GC content (GC) and in codon positions 1 (GC1), 2 (GC2) and 3 (GC3) (in percentage).

The dinucleotide odds ratio (*Rho)* was computed for each dinucleotide pair using the R library *seqinr*^46^.

Briefly, the *Rho* represents the frequency of dinucleotide (xy) divided by the product of frequencies of nucleotide (x) and nucleotide (y) and it is expected to be equal to 1.00 when dinucleotide (xy) is formed by chance. Since dinucleotide frequencies can be biased by the protein primary structure (i.e. amino acid sequence) and codon usage bias of these genes, the observed *Rho* was normalized by its expectation and variance estimated through random sequence generation, thus allowing to evaluate the degree of over- under-representation and its statistical significance. Particularly, the selected models allowed for random sequence generation by shuffling of synonymous codons, without affecting the codon usage bias and protein structure. A total of 1000 simulated sequences were generated for each dinucleotide pair.

### Relative synonymous codon usage (RSCU) and effective number of codons (Nc)

The RSCU was calculated using the *seqinr* package in R. This statistic, indicative of codon bias, is calculated based on the number of times a particular codon is observed, relative to the number of times that the codon would be observed assuming a uniform synonymous codon usage. Consequently, in the absence of any codon bias, a value close to 1 is expected, while synonymous codons with values lower than 0.6 or greater than 1.6 are regarded as under- or over-represented, respectively^24,27^.

The Nc values were calculated using the http://agnigarh.tezu.ernet.ihn/~ssankar/cub.php website. This summary statistic describes the total number of different codons used in a sequence and can thus range between 21 (only one codon used for each amino-acid) and 60 (all synonymous codons are uniformly used). A second parameter, the Nc′ statistic (also ranging between 21 and 60) was calculated to account for the effect of genome composition on codon bias. Obtained Nc and Nc′ values were plotted against their GC3 content and compared with the expected Nc distribution under the assumption that it is determined only by GC3 content, similarly to what performed in Franzo et al. (2017, 2018)^24,25^.

Additionally, the effective number of codons of the human lung genes was also evaluated. To this purpose, human lung protein expression levels were obtained from Wang et al.^47^ through the Expression atlas of EMBL-EBI interface^48^. Proteins in the upper 25% expression quantile were selected and the relative coding sequences were downloaded and analyzed as previously described.

### Principal component analysis (PCA)

The principal component analysis was performed independently on the dinucleotide Zscore and RSCU of all genes, after centering and scaling, using the prcomp function of the *stats* library in R.

All images were created using the *ggplot2*^49^ library in R.

## Supplementary Information

Supplementary Information.

## Data Availability

All sequences used in the present manuscript are freely available in Genbank. The specific datasets generated during the current study are available from the corresponding author upon request.

## Code availability

All analyses and computation were performed using previously developed and published R and Python libraries. The scripts for data pre-processing and manipulation are available from the corresponding author upon request.
