# Supplementary material for: SARS-CoV-2 and other human coronavirus show genome patterns previously associated to reduced viral recognition and altered immune response
Source: Sci Rep. 2021 May 21;11:10696. doi: 10.1038/s41598-021-90278-4 (PMC8139983; doi:10.1038/s41598-021-90278-4)
Supplement: Supplementary file 1 — Supplementary Information. [file 41598_2021_90278_MOESM1_ESM.pdf]

SARS-CoV-2 and other human coronavirus show genome patterns previously associated to reduced viral recognition and altered immune response

Giovanni Franzo[1*]

[1] *Department of Animal Medicine, Production and Health (MAPS), University of Padua, Legnaro, Padua, Italy*

Supplementary table 1

pp1ab: Summary of different pp1av CDS composition statistics calculated for each HCoV species The P-value refers to the presence of a significant difference in the mean value of the considered statistic among species

	HCOV-OC43 (N = 759)	HCOV-229E (N = 201)	HCOV-HKU1 (N = 97)	HCOV-NL63 (N = 367)	MERS-COV (N = 1,358)	SARS-COV (N = 540)	SARS-COV-2 (N = 834)	P-VALUE
**A**								
**MINIMUM**	0·236	0·255	0·209	0·222	0·236	0·226	0·215	
**MAXIMUM**	0·300	0·304	0·307	0·270	0·298	0·317	0·319	
**MEDIAN (IQR)**	0·29 (0·27, 0·30)	0·30 (0·27, 0·30)	0·28 (0·25, 0·29)	0·27 (0·26, 0·27)	0·26 (0·25, 0·26)	0·28 (0·25, 0·28)	0·29 (0·26, 0·30)	
**MEAN ± SD**	0·284 ± 0·016	0·288 ± 0·018	0·273 ± 0·021	0·260 ± 0·013	0·263 ± 0·015	0·272 ± 0·029	0·277 ± 0·037	P < 0·0001
**C**								
**MINIMUM**	0·118	0·157	0·112	0·133	0·191	0·194	0·175	
**MAXIMUM**	0·224	0·224	0·229	0·208	0·260	0·264	0·251	
**MEDIAN (IQR)**	0·16 (0·14, 0·22)	0·22 (0·18, 0·22)	0·14 (0·13, 0·21)	0·17 (0·16, 0·20)	0·21 (0·19, 0·22)	0·20 (0·20, 0·22)	0·20 (0·19, 0·22)	
**MEAN ± SD**	0·177 ± 0·035	0·204 ± 0·024	0·165 ± 0·040	0·178 ± 0·026	0·214 ± 0·022	0·217 ± 0·025	0·207 ± 0·026	P < 0·0001
**G**								
**MINIMUM**	0·187	0·162	0·153	0·141	0·189	0·186	0·184	
**MAXIMUM**	0·244	0·219	0·200	0·217	0·218	0·236	0·230	
**MEDIAN (IQR)**	0·22 (0·19, 0·24)	0·21 (0·21, 0·21)	0·19 (0·17, 0·19)	0·21 (0·18, 0·21)	0·21 (0·19, 0·22)	0·21 (0·20, 0·22)	0·20 (0·18, 0·21)	
**MEAN ± SD**	0·217 ± 0·020	0·211 ± 0·010	0·179 ± 0·014	0·195 ± 0·022	0·207 ± 0·010	0·209 ± 0·016	0·200 ± 0·015	P < 0·0001
**T**								
**MINIMUM**	0·237	0·260	0·281	0·318	0·229	0·199	0·210	
**MAXIMUM**	0·422	0·380	0·524	0·460	0·349	0·368	0·408	
**MEDIAN (IQR)**	0·36 (0·24, 0·36)	0·26 (0·26, 0·35)	0·40 (0·31, 0·43)	0·39 (0·32, 0·40)	0·33 (0·32, 0·34)	0·31 (0·30, 0·33)	0·32 (0·32, 0·33)	
**MEAN ± SD**	0·322 ± 0·063	0·297 ± 0·044	0·383 ± 0·066	0·367 ± 0·042	0·316 ± 0·038	0·302 ± 0·056	0·317 ± 0·063	P < 0·0001
**GC**								

**MINIMUM**	0·337	0·329	0·267	0·295	0·394	0·386	0·373	
**MAXIMUM**	0·466	0·438	0·415	0·416	0·475	0·485	0·473	
**MEDIAN (IQR)**	0·36 (0·36, 0·46)	0·43 (0·39, 0·44)	0·32 (0·31, 0·40)	0·37 (0·35, 0·41)	0·41 (0·41, 0·43)	0·41 (0·40, 0·45)	0·38 (0·37, 0·43)	
**MEAN ± SD**	0·394 ± 0·051	0·414 ± 0·029	0·344 ± 0·047	0·373 ± 0·038	0·421 ± 0·025	0·426 ± 0·036	0·407 ± 0·039	P < 0·0001
**GC1**								
**MINIMUM**	0·346	0·434	0·337	0·368	0·434	0·456	0·443	
**MAXIMUM**	0·526	0·554	0·489	0·513	0·549	0·556	0·536	
**MEDIAN (IQR)**	0·46 (0·43, 0·52)	0·55 (0·45, 0·55)	0·43 (0·37, 0·48)	0·45 (0·43, 0·51)	0·48 (0·45, 0·48)	0·49 (0·49, 0·50)	0·47 (0·47, 0·48)	
**MEAN ± SD**	0·460 ± 0·053	0·511 ± 0·050	0·420 ± 0·057	0·465 ± 0·040	0·482 ± 0·031	0·498 ± 0·031	0·483 ± 0·028	P < 0·0001
**GC2**								
**MINIMUM**	0·282	0·218	0·297	0·158	0·361	0·325	0·329	
**MAXIMUM**	0·479	0·445	0·511	0·431	0·490	0·506	0·502	
**MEDIAN (IQR)**	0·39 (0·36, 0·47)	0·44 (0·41, 0·44)	0·39 (0·34, 0·46)	0·40 (0·37, 0·42)	0·42 (0·39, 0·42)	0·40 (0·39, 0·41)	0·40 (0·38, 0·41)	
**MEAN ± SD**	0·402 ± 0·057	0·414 ± 0·045	0·396 ± 0·066	0·385 ± 0·058	0·411 ± 0·039	0·404 ± 0·059	0·405 ± 0·057	P < 0·0001
**GC3**								
**MINIMUM**	0·234	0·292	0·129	0·200	0·317	0·300	0·266	
**MAXIMUM**	0·403	0·425	0·281	0·405	0·436	0·486	0·459	
**MEDIAN (IQR)**	0·31 (0·25, 0·40)	0·32 (0·31, 0·32)	0·23 (0·18, 0·24)	0·28 (0·24, 0·30)	0·36 (0·36, 0·39)	0·39 (0·35, 0·39)	0·34 (0·27, 0·38)	
**MEAN ± SD**	0·319 ± 0·066	0·318 ± 0·023	0·215 ± 0·037	0·268 ± 0·038	0·370 ± 0·034	0·378 ± 0·054	0·332 ± 0·052	P < 0·0001

E: Summary of different E CDS composition statistics calculated for each HCoV species The P-value refers to the presence of a significant difference in the mean value of the considered statistic among species

	HCOV-OC43 (N = 759)	HCOV-229E (N = 201)	HCOV-HKU1 (N = 97)	HCOV-NL63 (N = 367)	MERS-COV (N = 1,358)	SARS-COV (N = 540)	SARS-COV-2 (N = 834)	P-VALUE
**A**								
**MINIMUM**	0·236	0·255	0·209	0·222	0·236	0·226	0·215	
**MAXIMUM**	0·300	0·304	0·307	0·270	0·298	0·317	0·319	
**MEDIAN (IQR)**	0·29 (0·27, 0·30)	0·30 (0·27, 0·30)	0·28 (0·25, 0·29)	0·27 (0·26, 0·27)	0·26 (0·25, 0·26)	0·28 (0·25, 0·28)	0·29 (0·26, 0·30)	
**MEAN ± SD**	0·284 ± 0·016	0·288 ± 0·018	0·273 ± 0·021	0·260 ± 0·013	0·263 ± 0·015	0·272 ± 0·029	0·277 ± 0·037	P < 0·0001
**C**								
**MINIMUM**	0·118	0·157	0·112	0·133	0·191	0·194	0·175	
**MAXIMUM**	0·224	0·224	0·229	0·208	0·260	0·264	0·251	
**MEDIAN (IQR)**	0·16 (0·14, 0·22)	0·22 (0·18, 0·22)	0·14 (0·13, 0·21)	0·17 (0·16, 0·20)	0·21 (0·19, 0·22)	0·20 (0·20, 0·22)	0·20 (0·19, 0·22)	
**MEAN ± SD**	0·177 ± 0·035	0·204 ± 0·024	0·165 ± 0·040	0·178 ± 0·026	0·214 ± 0·022	0·217 ± 0·025	0·207 ± 0·026	P < 0·0001
**G**								
**MINIMUM**	0·187	0·162	0·153	0·141	0·189	0·186	0·184	
**MAXIMUM**	0·244	0·219	0·200	0·217	0·218	0·236	0·230	
**MEDIAN (IQR)**	0·22 (0·19, 0·24)	0·21 (0·21, 0·21)	0·19 (0·17, 0·19)	0·21 (0·18, 0·21)	0·21 (0·19, 0·22)	0·21 (0·20, 0·22)	0·20 (0·18, 0·21)	
**MEAN ± SD**	0·217 ± 0·020	0·211 ± 0·010	0·179 ± 0·014	0·195 ± 0·022	0·207 ± 0·010	0·209 ± 0·016	0·200 ± 0·015	P < 0·0001
**T**								
**MINIMUM**	0·237	0·260	0·281	0·318	0·229	0·199	0·210	
**MAXIMUM**	0·422	0·380	0·524	0·460	0·349	0·368	0·408	
**MEDIAN (IQR)**	0·36 (0·24, 0·36)	0·26 (0·26, 0·35)	0·40 (0·31, 0·43)	0·39 (0·32, 0·40)	0·33 (0·32, 0·34)	0·31 (0·30, 0·33)	0·32 (0·32, 0·33)	
**MEAN ± SD**	0·322 ± 0·063	0·297 ± 0·044	0·383 ± 0·066	0·367 ± 0·042	0·316 ± 0·038	0·302 ± 0·056	0·317 ± 0·063	P < 0·0001
**GC**								

**MINIMUM**	0·337	0·329	0·267	0·295	0·394	0·386	0·373	
**MAXIMUM**	0·466	0·438	0·415	0·416	0·475	0·485	0·473	
**MEDIAN (IQR)**	0·36 (0·36, 0·46)	0·43 (0·39, 0·44)	0·32 (0·31, 0·40)	0·37 (0·35, 0·41)	0·41 (0·41, 0·43)	0·41 (0·40, 0·45)	0·38 (0·37, 0·43)	
**MEAN ± SD**	0·394 ± 0·051	0·414 ± 0·029	0·344 ± 0·047	0·373 ± 0·038	0·421 ± 0·025	0·426 ± 0·036	0·407 ± 0·039	P < 0·0001
**GC1**								
**MINIMUM**	0·346	0·434	0·337	0·368	0·434	0·456	0·443	
**MAXIMUM**	0·526	0·554	0·489	0·513	0·549	0·556	0·536	
**MEDIAN (IQR)**	0·46 (0·43, 0·52)	0·55 (0·45, 0·55)	0·43 (0·37, 0·48)	0·45 (0·43, 0·51)	0·48 (0·45, 0·48)	0·49 (0·49, 0·50)	0·47 (0·47, 0·48)	
**MEAN ± SD**	0·460 ± 0·053	0·511 ± 0·050	0·420 ± 0·057	0·465 ± 0·040	0·482 ± 0·031	0·498 ± 0·031	0·483 ± 0·028	P < 0·0001
**GC2**								
**MINIMUM**	0·282	0·218	0·297	0·158	0·361	0·325	0·329	
**MAXIMUM**	0·479	0·445	0·511	0·431	0·490	0·506	0·502	
**MEDIAN (IQR)**	0·39 (0·36, 0·47)	0·44 (0·41, 0·44)	0·39 (0·34, 0·46)	0·40 (0·37, 0·42)	0·42 (0·39, 0·42)	0·40 (0·39, 0·41)	0·40 (0·38, 0·41)	
**MEAN ± SD**	0·402 ± 0·057	0·414 ± 0·045	0·396 ± 0·066	0·385 ± 0·058	0·411 ± 0·039	0·404 ± 0·059	0·405 ± 0·057	P < 0·0001
**GC3**								
**MINIMUM**	0·234	0·292	0·129	0·200	0·317	0·300	0·266	
**MAXIMUM**	0·403	0·425	0·281	0·405	0·436	0·486	0·459	
**MEDIAN (IQR)**	0·31 (0·25, 0·40)	0·32 (0·31, 0·32)	0·23 (0·18, 0·24)	0·28 (0·24, 0·30)	0·36 (0·36, 0·39)	0·39 (0·35, 0·39)	0·34 (0·27, 0·38)	
**MEAN ± SD**	0·319 ± 0·066	0·318 ± 0·023	0·215 ± 0·037	0·268 ± 0·038	0·370 ± 0·034	0·378 ± 0·054	0·332 ± 0·052	P < 0·0001

M: Summary of different M CDS composition statistics calculated for each HCoV species The P-value refers to the presence of a significant difference in the mean value of the considered statistic among species

	HCOV-OC43 (N = 759)	HCOV-229E (N = 201)	HCOV-HKU1 (N = 97)	HCOV-NL63 (N = 367)	MERS-COV (N = 1,358)	SARS-COV (N = 540)	SARS-COV-2 (N = 834)	P-VALUE
**A**								
**MINIMUM**	0·236	0·255	0·209	0·222	0·236	0·226	0·215	
**MAXIMUM**	0·300	0·304	0·307	0·270	0·298	0·317	0·319	
**MEDIAN (IQR)**	0·29 (0·27, 0·30)	0·30 (0·27, 0·30)	0·28 (0·25, 0·29)	0·27 (0·26, 0·27)	0·26 (0·25, 0·26)	0·28 (0·25, 0·28)	0·29 (0·26, 0·30)	
**MEAN ± SD**	0·284 ± 0·016	0·288 ± 0·018	0·273 ± 0·021	0·260 ± 0·013	0·263 ± 0·015	0·272 ± 0·029	0·277 ± 0·037	P < 0·0001
**C**								
**MINIMUM**	0·118	0·157	0·112	0·133	0·191	0·194	0·175	
**MAXIMUM**	0·224	0·224	0·229	0·208	0·260	0·264	0·251	
**MEDIAN (IQR)**	0·16 (0·14, 0·22)	0·22 (0·18, 0·22)	0·14 (0·13, 0·21)	0·17 (0·16, 0·20)	0·21 (0·19, 0·22)	0·20 (0·20, 0·22)	0·20 (0·19, 0·22)	
**MEAN ± SD**	0·177 ± 0·035	0·204 ± 0·024	0·165 ± 0·040	0·178 ± 0·026	0·214 ± 0·022	0·217 ± 0·025	0·207 ± 0·026	P < 0·0001
**G**								
**MINIMUM**	0·187	0·162	0·153	0·141	0·189	0·186	0·184	
**MAXIMUM**	0·244	0·219	0·200	0·217	0·218	0·236	0·230	
**MEDIAN (IQR)**	0·22 (0·19, 0·24)	0·21 (0·21, 0·21)	0·19 (0·17, 0·19)	0·21 (0·18, 0·21)	0·21 (0·19, 0·22)	0·21 (0·20, 0·22)	0·20 (0·18, 0·21)	
**MEAN ± SD**	0·217 ± 0·020	0·211 ± 0·010	0·179 ± 0·014	0·195 ± 0·022	0·207 ± 0·010	0·209 ± 0·016	0·200 ± 0·015	P < 0·0001
**T**								
**MINIMUM**	0·237	0·260	0·281	0·318	0·229	0·199	0·210	
**MAXIMUM**	0·422	0·380	0·524	0·460	0·349	0·368	0·408	
**MEDIAN (IQR)**	0·36 (0·24, 0·36)	0·26 (0·26, 0·35)	0·40 (0·31, 0·43)	0·39 (0·32, 0·40)	0·33 (0·32, 0·34)	0·31 (0·30, 0·33)	0·32 (0·32, 0·33)	
**MEAN ± SD**	0·322 ± 0·063	0·297 ± 0·044	0·383 ± 0·066	0·367 ± 0·042	0·316 ± 0·038	0·302 ± 0·056	0·317 ± 0·063	P < 0·0001
**GC**								
**MINIMUM**	0·337	0·329	0·267	0·295	0·394	0·386	0·373	
**MAXIMUM**	0·466	0·438	0·415	0·416	0·475	0·485	0·473	

**MEDIAN (IQR)**	0·36 (0·36, 0·46)	0·43 (0·39, 0·44)	0·32 (0·31, 0·40)	0·37 (0·35, 0·41)	0·41 (0·41, 0·43)	0·41 (0·40, 0·45)	0·38 (0·37, 0·43)	
**MEAN ± SD**	0·394 ± 0·051	0·414 ± 0·029	0·344 ± 0·047	0·373 ± 0·038	0·421 ± 0·025	0·426 ± 0·036	0·407 ± 0·039	P < 0·0001
**GC1**								
**MINIMUM**	0·346	0·434	0·337	0·368	0·434	0·456	0·443	
**MAXIMUM**	0·526	0·554	0·489	0·513	0·549	0·556	0·536	
**MEDIAN (IQR)**	0·46 (0·43, 0·52)	0·55 (0·45, 0·55)	0·43 (0·37, 0·48)	0·45 (0·43, 0·51)	0·48 (0·45, 0·48)	0·49 (0·49, 0·50)	0·47 (0·47, 0·48)	
**MEAN ± SD**	0·460 ± 0·053	0·511 ± 0·050	0·420 ± 0·057	0·465 ± 0·040	0·482 ± 0·031	0·498 ± 0·031	0·483 ± 0·028	P < 0·0001
**GC2**								
**MINIMUM**	0·282	0·218	0·297	0·158	0·361	0·325	0·329	
**MAXIMUM**	0·479	0·445	0·511	0·431	0·490	0·506	0·502	
**MEDIAN (IQR)**	0·39 (0·36, 0·47)	0·44 (0·41, 0·44)	0·39 (0·34, 0·46)	0·40 (0·37, 0·42)	0·42 (0·39, 0·42)	0·40 (0·39, 0·41)	0·40 (0·38, 0·41)	
**MEAN ± SD**	0·402 ± 0·057	0·414 ± 0·045	0·396 ± 0·066	0·385 ± 0·058	0·411 ± 0·039	0·404 ± 0·059	0·405 ± 0·057	P < 0·0001
**GC3**								
**MINIMUM**	0·234	0·292	0·129	0·200	0·317	0·300	0·266	
**MAXIMUM**	0·403	0·425	0·281	0·405	0·436	0·486	0·459	
**MEDIAN (IQR)**	0·31 (0·25, 0·40)	0·32 (0·31, 0·32)	0·23 (0·18, 0·24)	0·28 (0·24, 0·30)	0·36 (0·36, 0·39)	0·39 (0·35, 0·39)	0·34 (0·27, 0·38)	
**MEAN ± SD**	0·319 ± 0·066	0·318 ± 0·023	0·215 ± 0·037	0·268 ± 0·038	0·370 ± 0·034	0·378 ± 0·054	0·332 ± 0·052	P < 0·0001

N: Summary of different N CDS composition statistics calculated for each HCoV species The P-value refers to the presence of a significant difference in the mean value of the considered statistic among species

	HCOV-OC43 (N = 759)	HCOV-229E (N = 201)	HCOV-HKU1 (N = 97)	HCOV-NL63 (N = 367)	MERS-COV (N = 1,358)	SARS-COV (N = 540)	SARS-COV-2 (N = 834)	P-VALUE
**A**								
**MINIMUM**	0·236	0·255	0·209	0·222	0·236	0·226	0·215	
**MAXIMUM**	0·300	0·304	0·307	0·270	0·298	0·317	0·319	
**MEDIAN (IQR)**	0·29 (0·27, 0·30)	0·30 (0·27, 0·30)	0·28 (0·25, 0·29)	0·27 (0·26, 0·27)	0·26 (0·25, 0·26)	0·28 (0·25, 0·28)	0·29 (0·26, 0·30)	
**MEAN ± SD**	0·284 ± 0·016	0·288 ± 0·018	0·273 ± 0·021	0·260 ± 0·013	0·263 ± 0·015	0·272 ± 0·029	0·277 ± 0·037	P < 0·0001
**C**								
**MINIMUM**	0·118	0·157	0·112	0·133	0·191	0·194	0·175	
**MAXIMUM**	0·224	0·224	0·229	0·208	0·260	0·264	0·251	
**MEDIAN (IQR)**	0·16 (0·14, 0·22)	0·22 (0·18, 0·22)	0·14 (0·13, 0·21)	0·17 (0·16, 0·20)	0·21 (0·19, 0·22)	0·20 (0·20, 0·22)	0·20 (0·19, 0·22)	
**MEAN ± SD**	0·177 ± 0·035	0·204 ± 0·024	0·165 ± 0·040	0·178 ± 0·026	0·214 ± 0·022	0·217 ± 0·025	0·207 ± 0·026	P < 0·0001
**G**								
**MINIMUM**	0·187	0·162	0·153	0·141	0·189	0·186	0·184	
**MAXIMUM**	0·244	0·219	0·200	0·217	0·218	0·236	0·230	
**MEDIAN (IQR)**	0·22 (0·19, 0·24)	0·21 (0·21, 0·21)	0·19 (0·17, 0·19)	0·21 (0·18, 0·21)	0·21 (0·19, 0·22)	0·21 (0·20, 0·22)	0·20 (0·18, 0·21)	
**MEAN ± SD**	0·217 ± 0·020	0·211 ± 0·010	0·179 ± 0·014	0·195 ± 0·022	0·207 ± 0·010	0·209 ± 0·016	0·200 ± 0·015	P < 0·0001
**T**								
**MINIMUM**	0·237	0·260	0·281	0·318	0·229	0·199	0·210	
**MAXIMUM**	0·422	0·380	0·524	0·460	0·349	0·368	0·408	
**MEDIAN (IQR)**	0·36 (0·24, 0·36)	0·26 (0·26, 0·35)	0·40 (0·31, 0·43)	0·39 (0·32, 0·40)	0·33 (0·32, 0·34)	0·31 (0·30, 0·33)	0·32 (0·32, 0·33)	
**MEAN ± SD**	0·322 ± 0·063	0·297 ± 0·044	0·383 ± 0·066	0·367 ± 0·042	0·316 ± 0·038	0·302 ± 0·056	0·317 ± 0·063	P < 0·0001
**GC**								
**MINIMUM**	0·337	0·329	0·267	0·295	0·394	0·386	0·373	
**MAXIMUM**	0·466	0·438	0·415	0·416	0·475	0·485	0·473	

**MEDIAN (IQR)**	0·36 (0·36, 0·46)	0·43 (0·39, 0·44)	0·32 (0·31, 0·40)	0·37 (0·35, 0·41)	0·41 (0·41, 0·43)	0·41 (0·40, 0·45)	0·38 (0·37, 0·43)	
**MEAN ± SD**	0·394 ± 0·051	0·414 ± 0·029	0·344 ± 0·047	0·373 ± 0·038	0·421 ± 0·025	0·426 ± 0·036	0·407 ± 0·039	P < 0·0001
**GC1**								
**MINIMUM**	0·346	0·434	0·337	0·368	0·434	0·456	0·443	
**MAXIMUM**	0·526	0·554	0·489	0·513	0·549	0·556	0·536	
**MEDIAN (IQR)**	0·46 (0·43, 0·52)	0·55 (0·45, 0·55)	0·43 (0·37, 0·48)	0·45 (0·43, 0·51)	0·48 (0·45, 0·48)	0·49 (0·49, 0·50)	0·47 (0·47, 0·48)	
**MEAN ± SD**	0·460 ± 0·053	0·511 ± 0·050	0·420 ± 0·057	0·465 ± 0·040	0·482 ± 0·031	0·498 ± 0·031	0·483 ± 0·028	P < 0·0001
**GC2**								
**MINIMUM**	0·282	0·218	0·297	0·158	0·361	0·325	0·329	
**MAXIMUM**	0·479	0·445	0·511	0·431	0·490	0·506	0·502	
**MEDIAN (IQR)**	0·39 (0·36, 0·47)	0·44 (0·41, 0·44)	0·39 (0·34, 0·46)	0·40 (0·37, 0·42)	0·42 (0·39, 0·42)	0·40 (0·39, 0·41)	0·40 (0·38, 0·41)	
**MEAN ± SD**	0·402 ± 0·057	0·414 ± 0·045	0·396 ± 0·066	0·385 ± 0·058	0·411 ± 0·039	0·404 ± 0·059	0·405 ± 0·057	P < 0·0001
**GC3**								
**MINIMUM**	0·234	0·292	0·129	0·200	0·317	0·300	0·266	
**MAXIMUM**	0·403	0·425	0·281	0·405	0·436	0·486	0·459	
**MEDIAN (IQR)**	0·31 (0·25, 0·40)	0·32 (0·31, 0·32)	0·23 (0·18, 0·24)	0·28 (0·24, 0·30)	0·36 (0·36, 0·39)	0·39 (0·35, 0·39)	0·34 (0·27, 0·38)	
**MEAN ± SD**	0·319 ± 0·066	0·318 ± 0·023	0·215 ± 0·037	0·268 ± 0·038	0·370 ± 0·034	0·378 ± 0·054	0·332 ± 0·052	P < 0·0001

S: Summary of different S CDS composition statistics calculated for each HCoV species The P-value refers to the presence of a significant difference in the mean value of the considered statistic among species

	HCOV-OC43 (N = 759)	HCOV-229E (N = 201)	HCOV-HKU1 (N = 97)	HCOV-NL63 (N = 367)	MERS-COV (N = 1,358)	SARS-COV (N = 540)	SARS-COV-2 (N = 834)	P-VALUE
**A**								
**MINIMUM**	0·236	0·255	0·209	0·222	0·236	0·226	0·215	
**MAXIMUM**	0·300	0·304	0·307	0·270	0·298	0·317	0·319	
**MEDIAN (IQR)**	0·29 (0·27, 0·30)	0·30 (0·27, 0·30)	0·28 (0·25, 0·29)	0·27 (0·26, 0·27)	0·26 (0·25, 0·26)	0·28 (0·25, 0·28)	0·29 (0·26, 0·30)	
**MEAN ± SD**	0·284 ± 0·016	0·288 ± 0·018	0·273 ± 0·021	0·260 ± 0·013	0·263 ± 0·015	0·272 ± 0·029	0·277 ± 0·037	P < 0·0001
**C**								
**MINIMUM**	0·118	0·157	0·112	0·133	0·191	0·194	0·175	
**MAXIMUM**	0·224	0·224	0·229	0·208	0·260	0·264	0·251	
**MEDIAN (IQR)**	0·16 (0·14, 0·22)	0·22 (0·18, 0·22)	0·14 (0·13, 0·21)	0·17 (0·16, 0·20)	0·21 (0·19, 0·22)	0·20 (0·20, 0·22)	0·20 (0·19, 0·22)	
**MEAN ± SD**	0·177 ± 0·035	0·204 ± 0·024	0·165 ± 0·040	0·178 ± 0·026	0·214 ± 0·022	0·217 ± 0·025	0·207 ± 0·026	P < 0·0001
**G**								
**MINIMUM**	0·187	0·162	0·153	0·141	0·189	0·186	0·184	
**MAXIMUM**	0·244	0·219	0·200	0·217	0·218	0·236	0·230	
**MEDIAN (IQR)**	0·22 (0·19, 0·24)	0·21 (0·21, 0·21)	0·19 (0·17, 0·19)	0·21 (0·18, 0·21)	0·21 (0·19, 0·22)	0·21 (0·20, 0·22)	0·20 (0·18, 0·21)	
**MEAN ± SD**	0·217 ± 0·020	0·211 ± 0·010	0·179 ± 0·014	0·195 ± 0·022	0·207 ± 0·010	0·209 ± 0·016	0·200 ± 0·015	P < 0·0001
**T**								
**MINIMUM**	0·237	0·260	0·281	0·318	0·229	0·199	0·210	
**MAXIMUM**	0·422	0·380	0·524	0·460	0·349	0·368	0·408	
**MEDIAN (IQR)**	0·36 (0·24, 0·36)	0·26 (0·26, 0·35)	0·40 (0·31, 0·43)	0·39 (0·32, 0·40)	0·33 (0·32, 0·34)	0·31 (0·30, 0·33)	0·32 (0·32, 0·33)	
**MEAN ± SD**	0·322 ± 0·063	0·297 ± 0·044	0·383 ± 0·066	0·367 ± 0·042	0·316 ± 0·038	0·302 ± 0·056	0·317 ± 0·063	P < 0·0001
**GC**								
**MINIMUM**	0·337	0·329	0·267	0·295	0·394	0·386	0·373	
**MAXIMUM**	0·466	0·438	0·415	0·416	0·475	0·485	0·473	

**MEDIAN (IQR)**	0·36 (0·36, 0·46)	0·43 (0·39, 0·44)	0·32 (0·31, 0·40)	0·37 (0·35, 0·41)	0·41 (0·41, 0·43)	0·41 (0·40, 0·45)	0·38 (0·37, 0·43)	
**MEAN ± SD**	0·394 ± 0·051	0·414 ± 0·029	0·344 ± 0·047	0·373 ± 0·038	0·421 ± 0·025	0·426 ± 0·036	0·407 ± 0·039	P < 0·0001
**GC1**								
**MINIMUM**	0·346	0·434	0·337	0·368	0·434	0·456	0·443	
**MAXIMUM**	0·526	0·554	0·489	0·513	0·549	0·556	0·536	
**MEDIAN (IQR)**	0·46 (0·43, 0·52)	0·55 (0·45, 0·55)	0·43 (0·37, 0·48)	0·45 (0·43, 0·51)	0·48 (0·45, 0·48)	0·49 (0·49, 0·50)	0·47 (0·47, 0·48)	
**MEAN ± SD**	0·460 ± 0·053	0·511 ± 0·050	0·420 ± 0·057	0·465 ± 0·040	0·482 ± 0·031	0·498 ± 0·031	0·483 ± 0·028	P < 0·0001
**GC2**								
**MINIMUM**	0·282	0·218	0·297	0·158	0·361	0·325	0·329	
**MAXIMUM**	0·479	0·445	0·511	0·431	0·490	0·506	0·502	
**MEDIAN (IQR)**	0·39 (0·36, 0·47)	0·44 (0·41, 0·44)	0·39 (0·34, 0·46)	0·40 (0·37, 0·42)	0·42 (0·39, 0·42)	0·40 (0·39, 0·41)	0·40 (0·38, 0·41)	
**MEAN ± SD**	0·402 ± 0·057	0·414 ± 0·045	0·396 ± 0·066	0·385 ± 0·058	0·411 ± 0·039	0·404 ± 0·059	0·405 ± 0·057	P < 0·0001
**GC3**								
**MINIMUM**	0·234	0·292	0·129	0·200	0·317	0·300	0·266	
**MAXIMUM**	0·403	0·425	0·281	0·405	0·436	0·486	0·459	
**MEDIAN (IQR)**	0·31 (0·25, 0·40)	0·32 (0·31, 0·32)	0·23 (0·18, 0·24)	0·28 (0·24, 0·30)	0·36 (0·36, 0·39)	0·39 (0·35, 0·39)	0·34 (0·27, 0·38)	
**MEAN ± SD**	0·319 ± 0·066	0·318 ± 0·023	0·215 ± 0·037	0·268 ± 0·038	0·370 ± 0·034	0·378 ± 0·054	0·332 ± 0·052	P < 0·0001

Supplementary figure 1

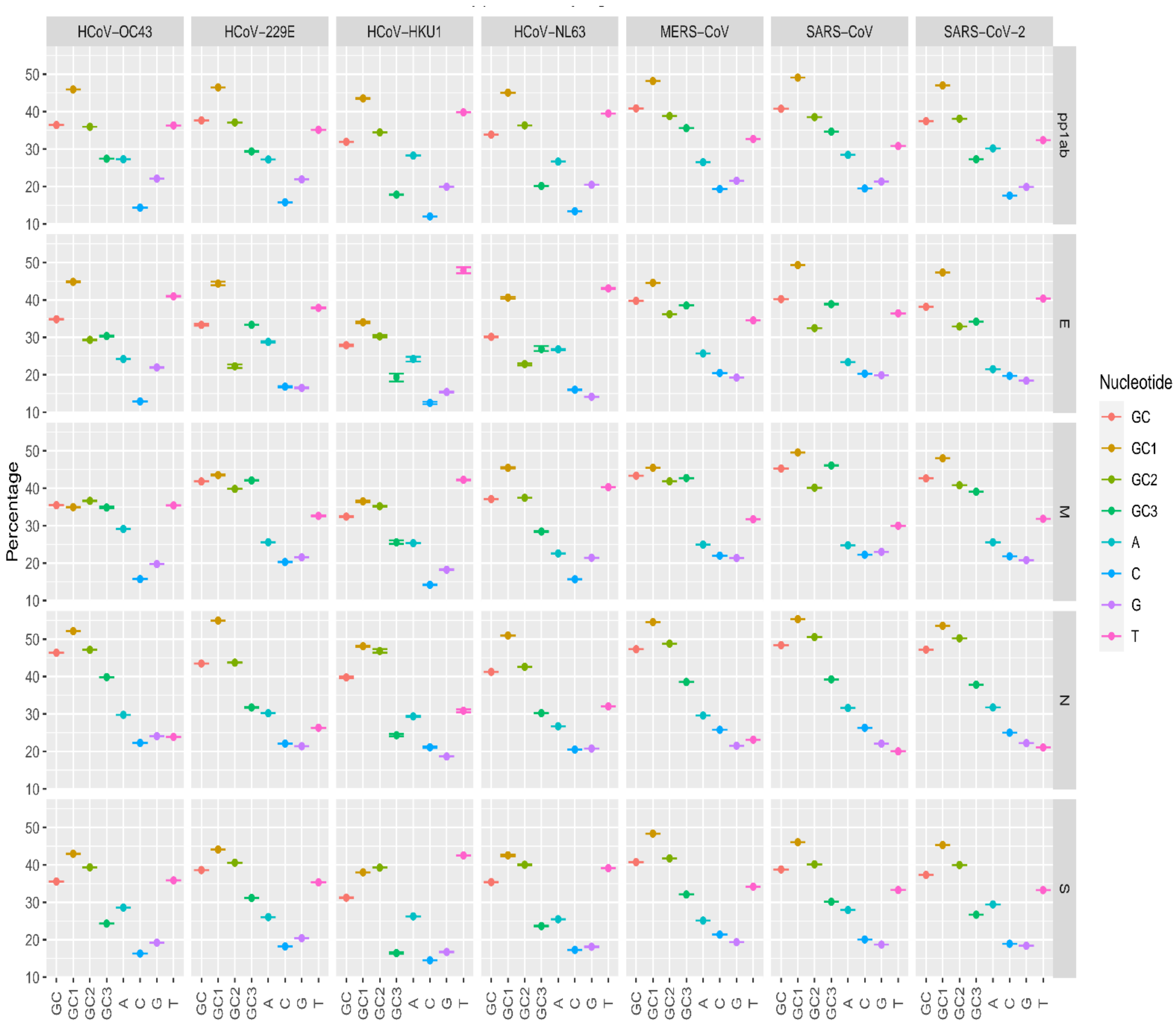

Mean (point) and 95% confidence interval (errorbar) of nucleotide content calculated for each gene−species pair.

Supplementary figure 2

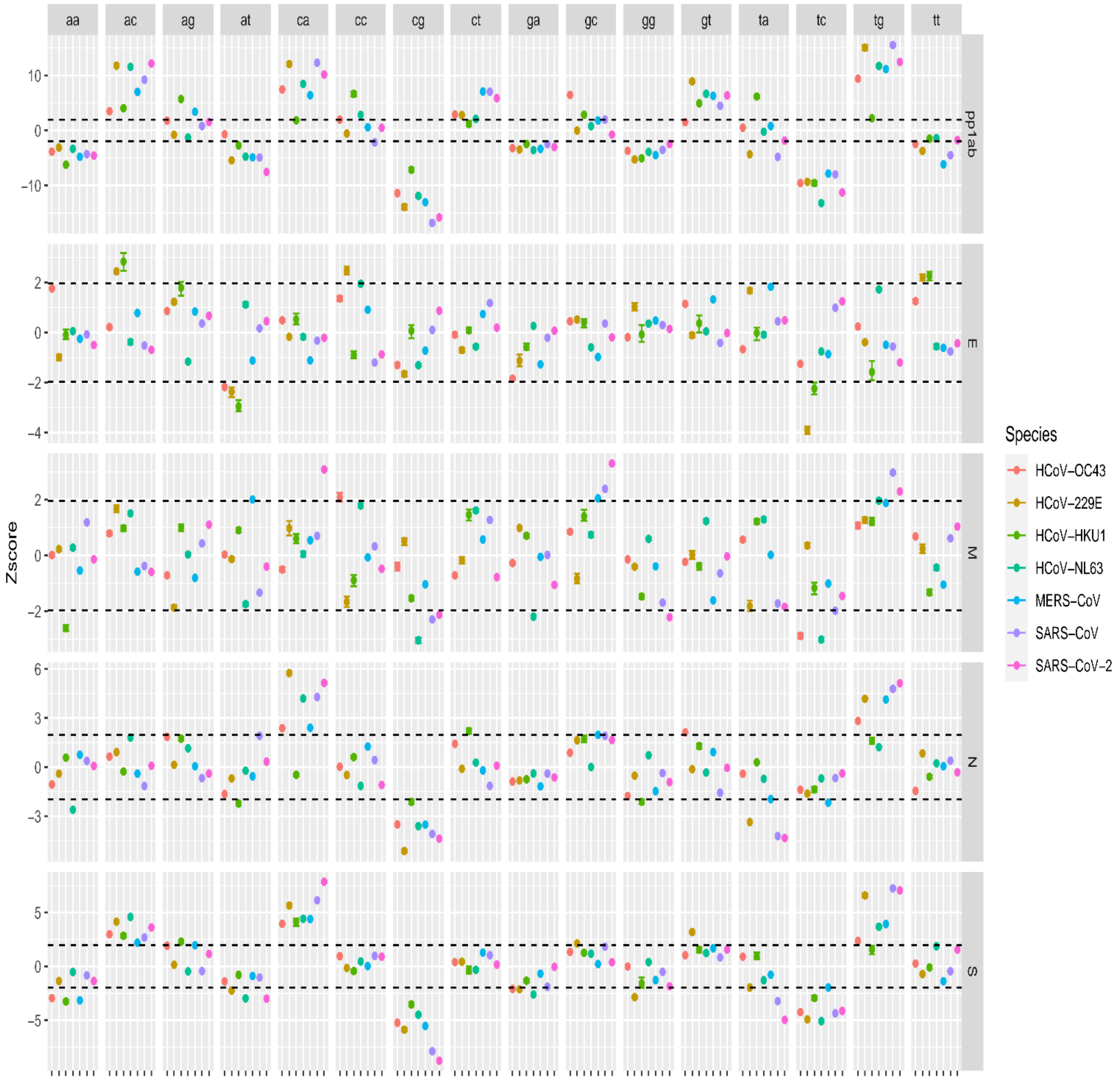

Mean (point) and 95% confidence interval (errorbar) calculated for the Zscore each dinucleotide-gene pair. The dashed lines (i.e. Zscore ±1.96) highlight the cut-off for significantly under- and overrepresented dinucleotides. Different gene-species combinations have been facetted.

Supplementary figure 3

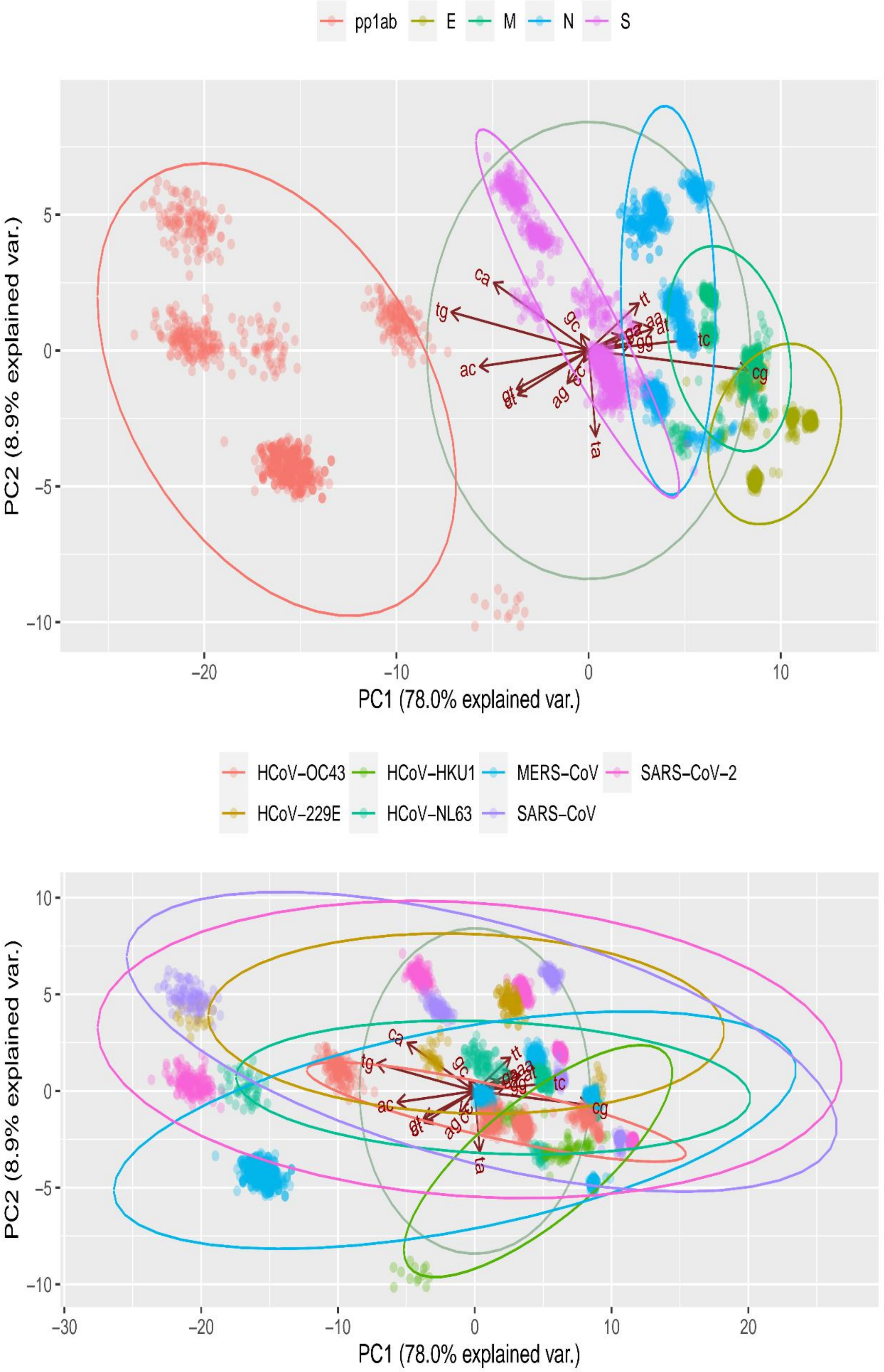

PCA based on Zscore. The individual sequences have been color coded based on the corresponding protein (upper figure) or viral species (lower figure). The PCA loadings are represented as arrows and the corresponding correlation circle has been reported. The 95% confidence ellipses around clusters are also reported.

Supplementary figure 4

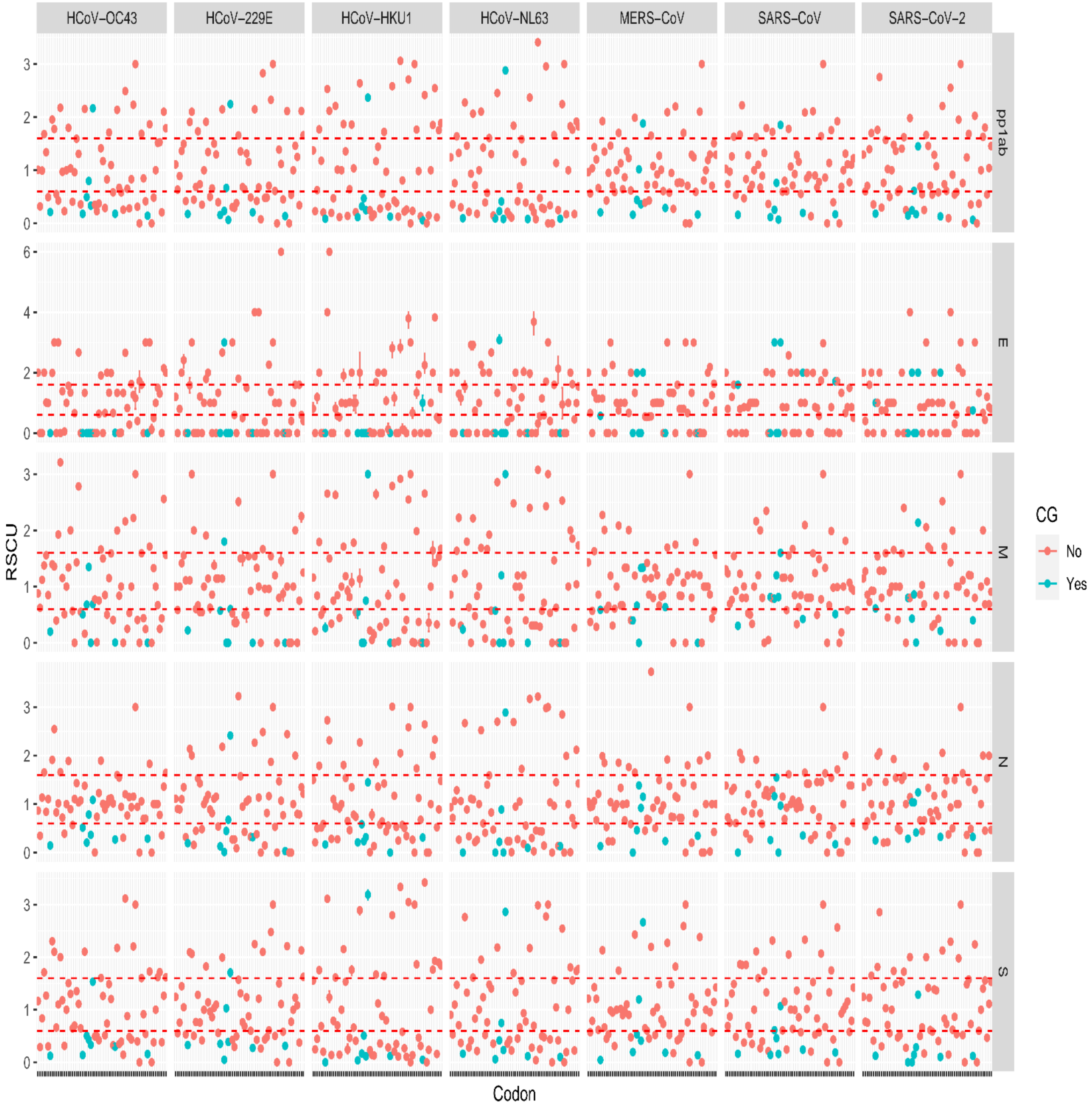

Mean (point) and 95% confidence intervals (error-bars) of relative synonymous codon usage (RSCU) are reported for each codon. For graphical reasons, codons labels have been removed. However, codons have been ordered alphabetically. Codons containing the CpG dinucleotide are highlighted in blue. Faceting has been used to display different gene-species combinations.

Supplementary figure 5

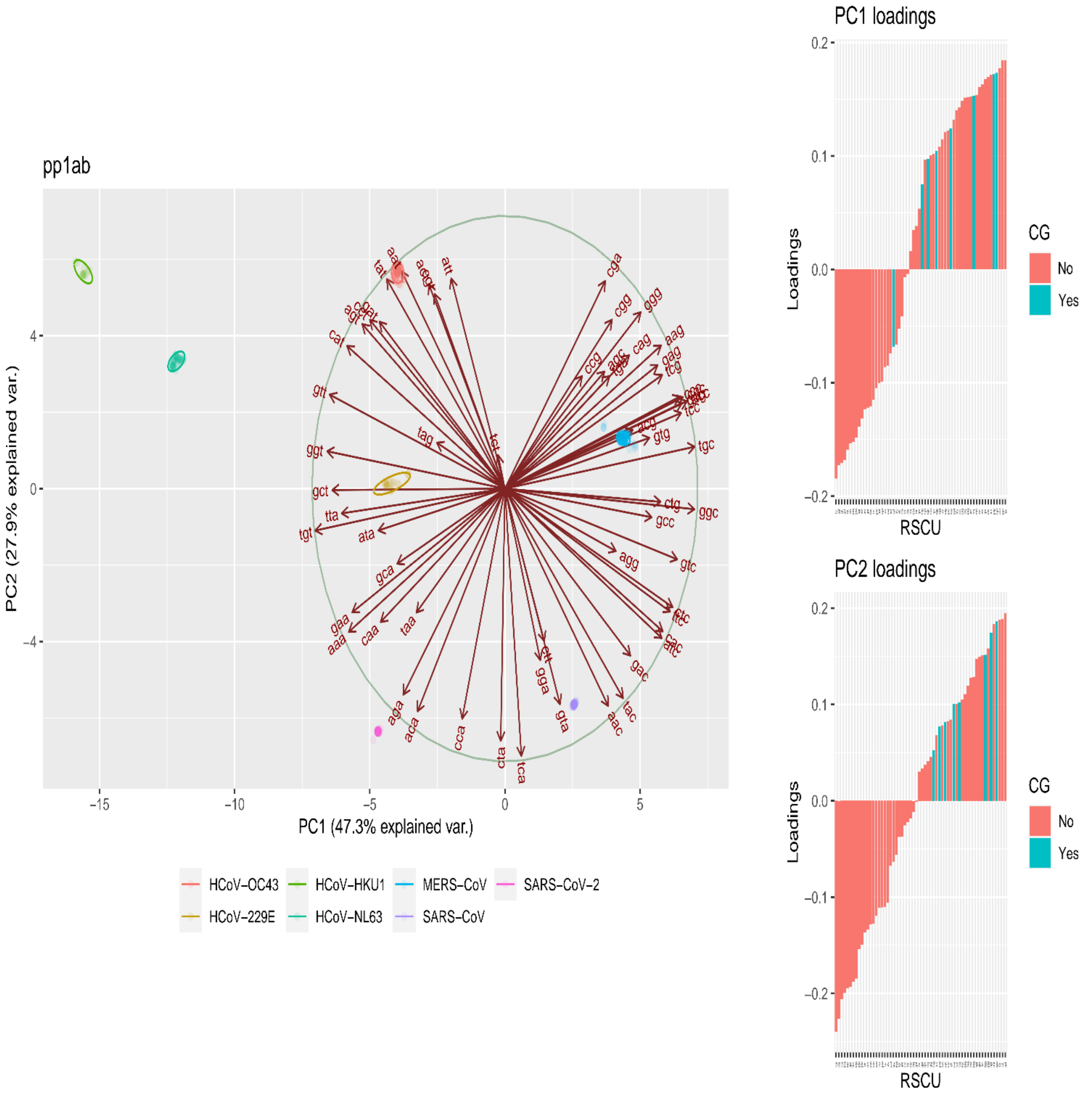

PCA based on relative synonymous codon usage of the pp1ab coding sequence. The individual sequences have been color coded based on the viral species. The PCA loadings are represented as arrows and the corresponding correlation circle has been reported. The 95% confidence ellipses around clusters are also reported. On the right inserts, the PC1 and 2 loadings are displayed. Codons displaying the CpG pair are highlighted in blue.

Supplementary figure 6

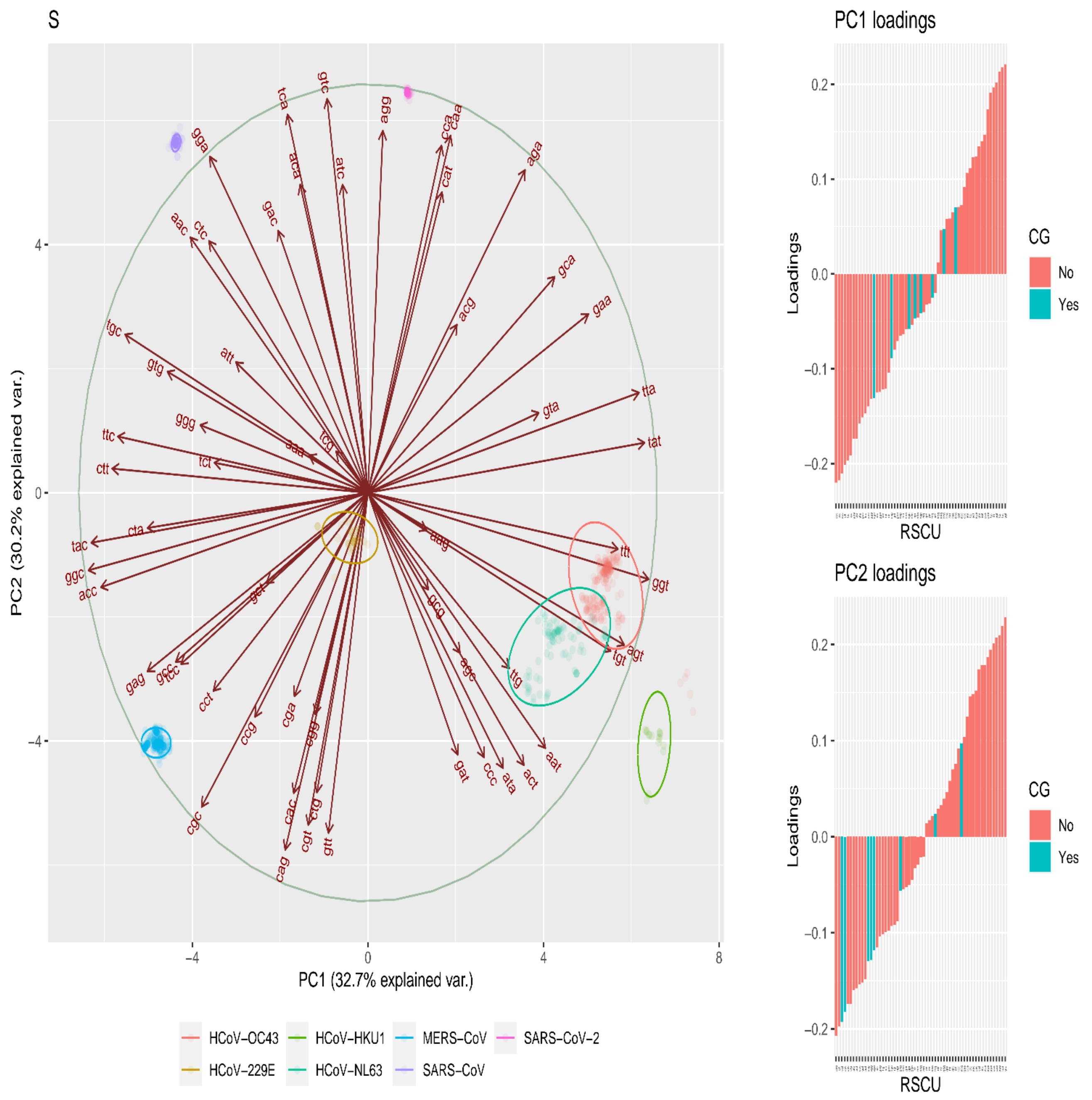

PCA based on relative synonymous codon usage of the spike coding sequence. The individual sequences have been color coded based on the viral species. The PCA loadings are represented as arrows and the corresponding correlation circle has been reported. The 95% confidence ellipses around clusters are also reported. On the right inserts, the PC1 and 2 loadings are displayed. Codons displaying the CpG pair are highlighted in blue.

Supplementary figure 7

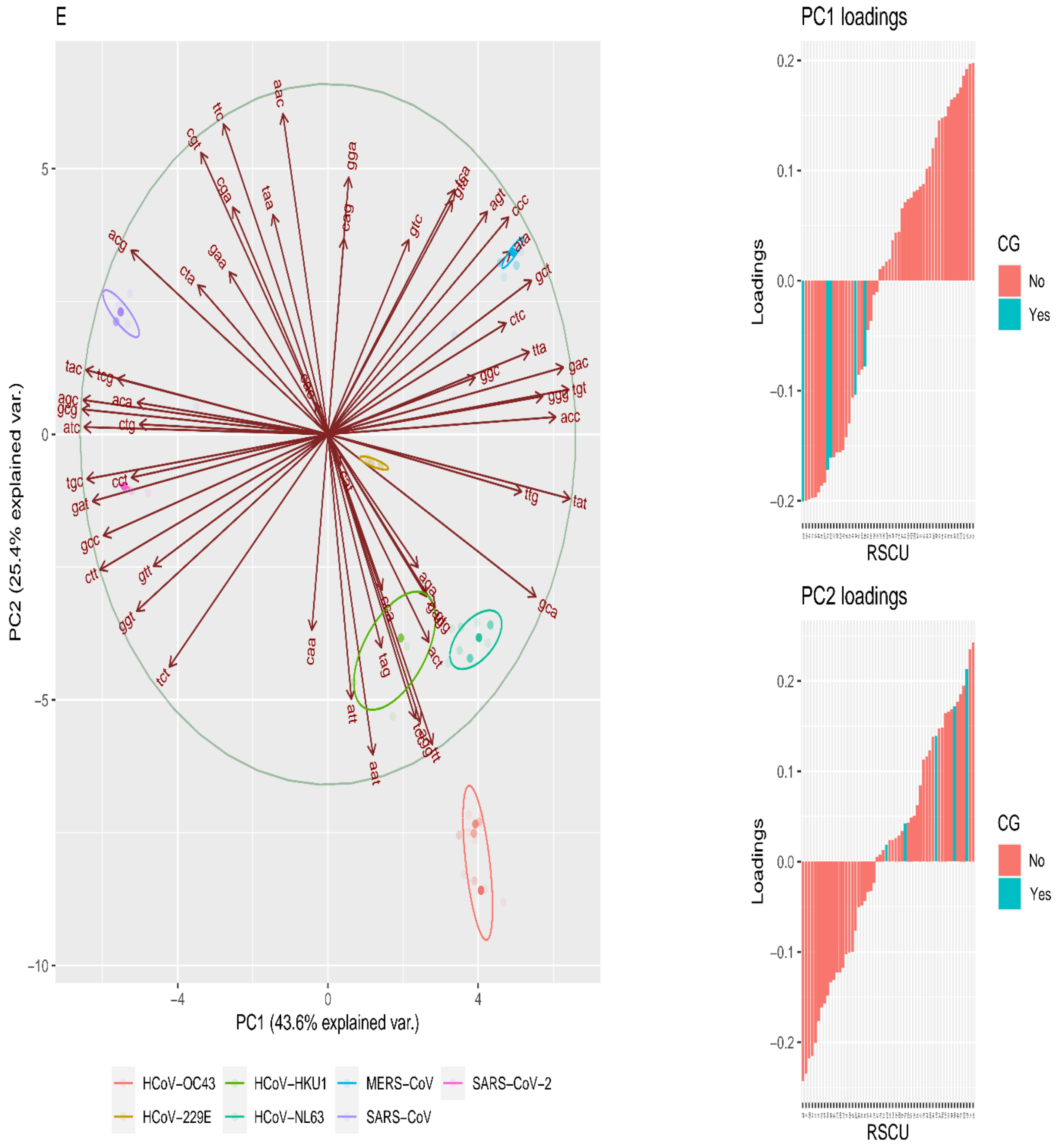

PCA based on relative synonymous codon usage of the envelope coding sequence. The individual sequences have been color coded based on the viral species. The PCA loadings are represented as arrows and the corresponding correlation circle has been reported. The 95% confidence ellipses around clusters are also reported. On the right inserts, the PC1 and 2 loadings are displayed. Codons displaying the CpG pair are highlighted in blue.

Supplementary figure 8

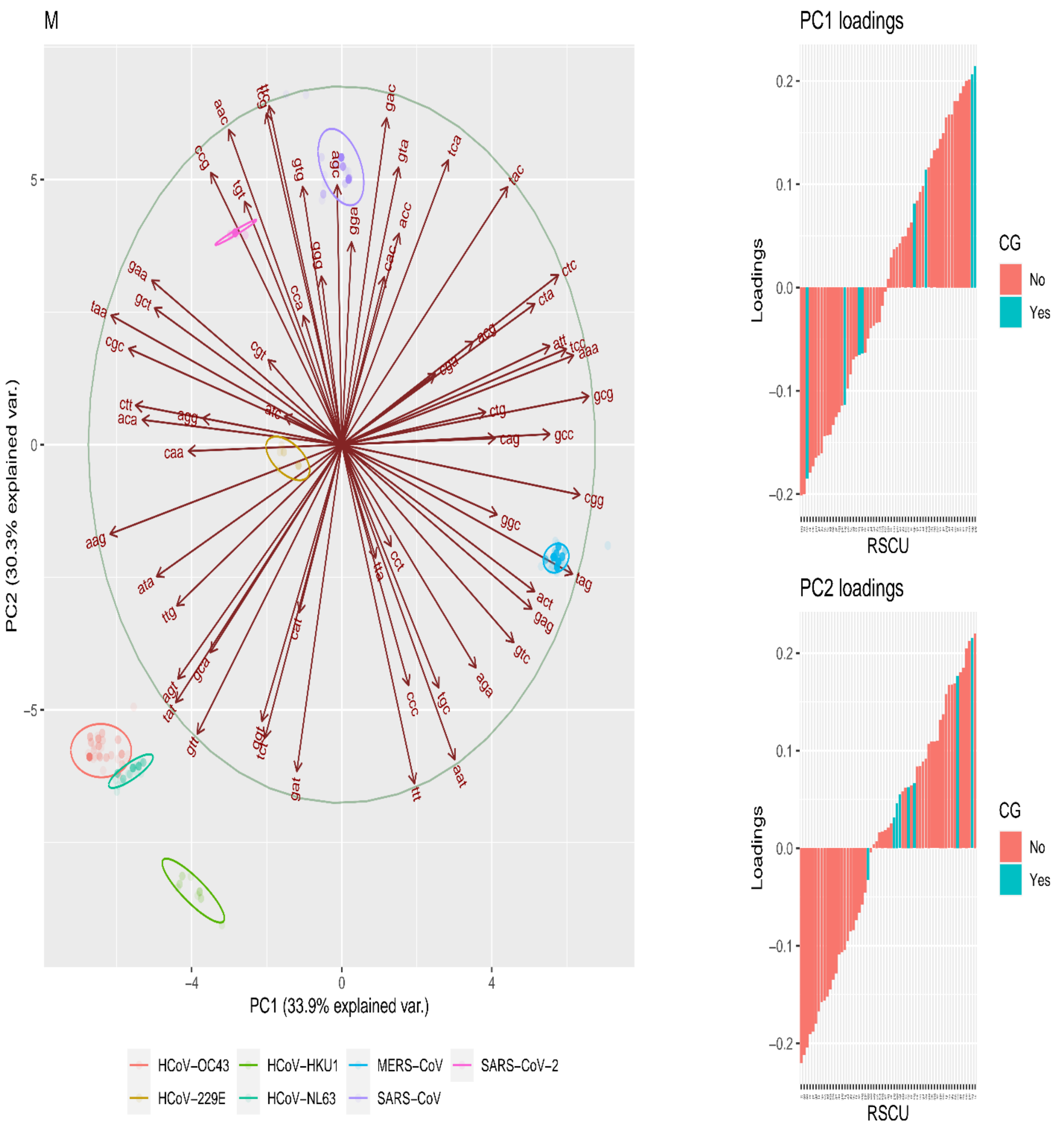

PCA based on relative synonymous codon usage of the matrix coding sequence. The individual sequences have been color coded based on the viral species. The PCA loadings are represented as arrows and the corresponding correlation circle has been reported. The 95% confidence ellipses around clusters are also reported. On the right inserts, the PC1 and 2 loadings are displayed. Codons displaying the CpG pair are highlighted in blue.

Supplementary figure 9

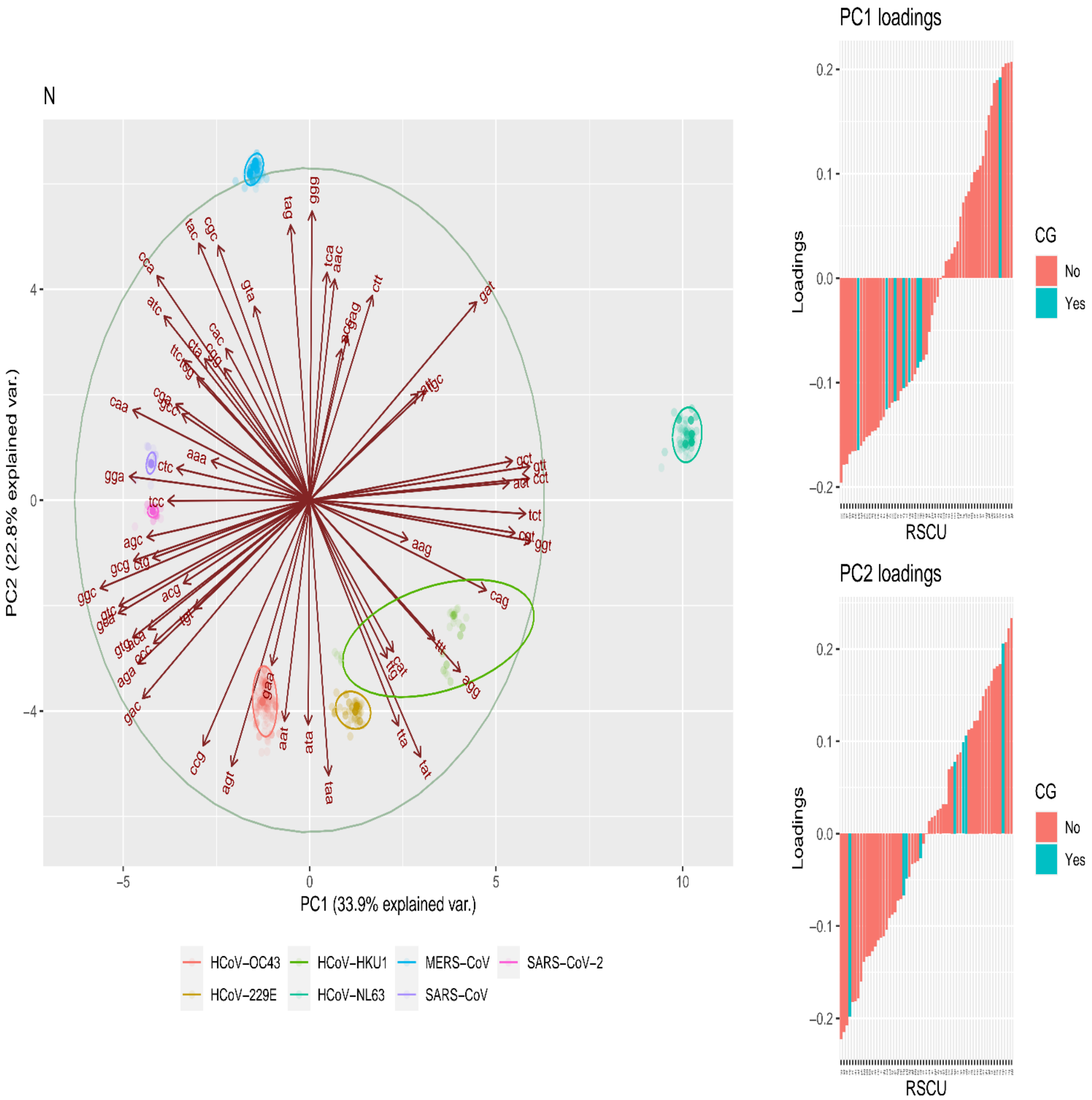

PCA based on relative synonymous codon usage of the nucleocapsid coding sequence. The individual sequences have been color coded based on the viral species. The PCA loadings are represented as arrows and the corresponding correlation circle has been reported. The 95% confidence ellipses around clusters are also reported. On the right inserts, the PC1 and 2 loadings are displayed. Codons displaying the CpG pair are highlighted in blue.

Supplementary figure 10

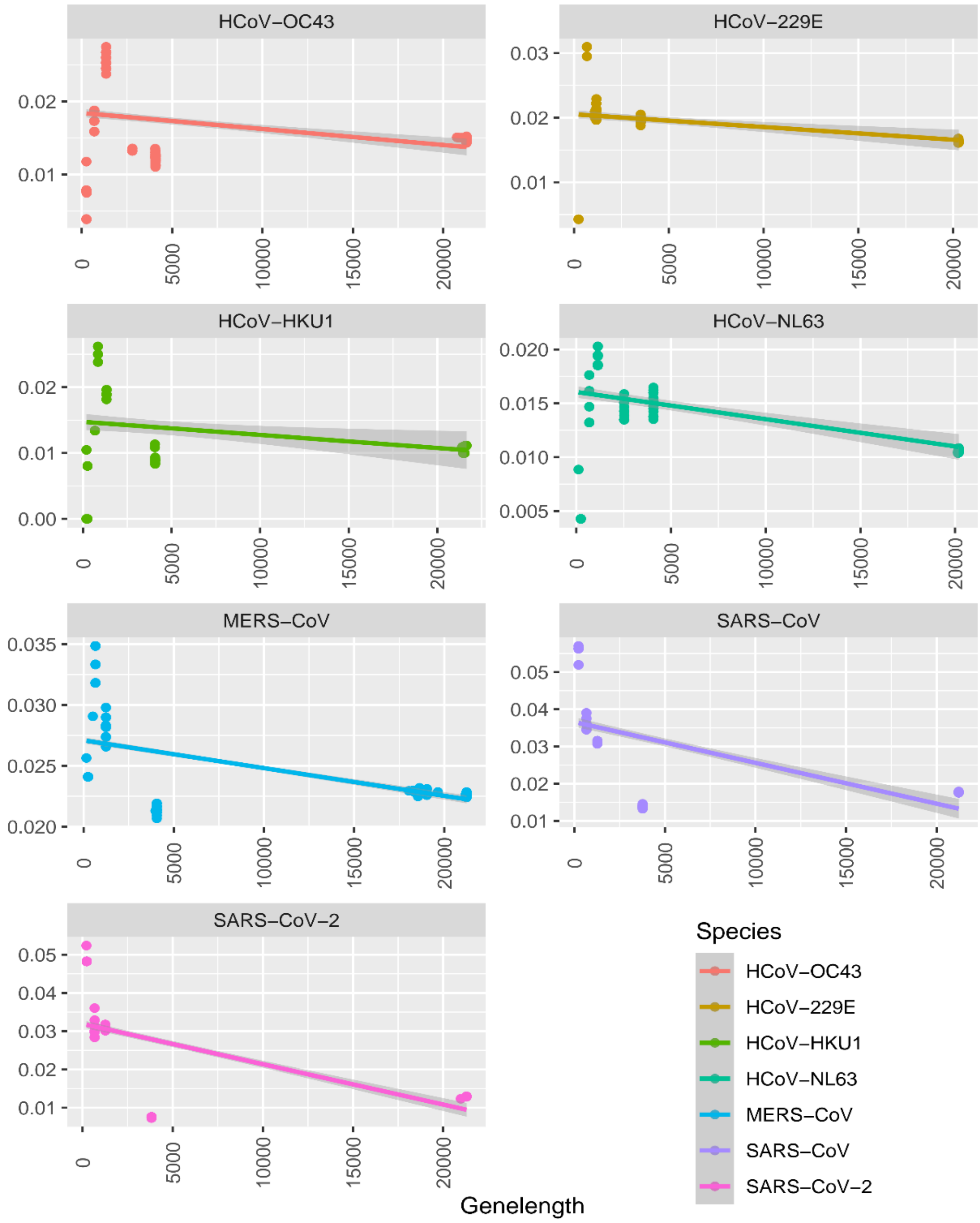

Relationship between CpG ratio (total number of CpG pairs within the gene divided by gene length) and gene length. Species have been faceted and color-coded. A linear regression line has been superimposed. The relative regression coefficients and significance level are provided in supplementary table 2.

Supplementary table 2

Species	Regression coefficient	P-value
**HCoV-OC43**	-2·19e-07	<0.001
**HCoV-229E**	-1·97e-07	<0.001
**HCoV-HKU1**	-1·99e-07	<0.001
**HCoV-NL63**	-2·53e-07	<0.001
**MERS-CoV**	-2·28e-07	<0.001
**SARS-CoV**	-1·10e-06	<0.001
**SARS-CoV-2**	-1·05e-06	<0.001

Coefficient and relative statistical significance of the linear regression between CpG ratio (total number of CpG pairs within the gene divided by gene length) and gene length (in bp). The linear models were fitted for each HCoV, independently.
